# Perfluorooctane sulfonate (PFOS) in follicular fluid and human granulosa cell dysfunction: a physiologically based toxicokinetic model translation of long-term low-level *in vitro* exposure data

**DOI:** 10.1093/hropen/hoag029

**Published:** 2026-04-07

**Authors:** Tamara Tomanic, Dragana Samardzija Nenadov, Sava Radovic Pletikosic, Bojana Stanic, Darija Obradovic, Sasa Lazovic, Nebojsa Andric

**Affiliations:** Department of Biology and Ecology, Faculty of Sciences, University of Novi Sad, Novi Sad, Serbia; Department of Biology and Ecology, Faculty of Sciences, University of Novi Sad, Novi Sad, Serbia; Department of Biology and Ecology, Faculty of Sciences, University of Novi Sad, Novi Sad, Serbia; Department of Biology and Ecology, Faculty of Sciences, University of Novi Sad, Novi Sad, Serbia; Institute of Physics Belgrade, National Institute of the Republic of Serbia, Belgrade, Serbia; Institute of Physics Belgrade, National Institute of the Republic of Serbia, Belgrade, Serbia; Department of Biology and Ecology, Faculty of Sciences, University of Novi Sad, Novi Sad, Serbia

**Keywords:** PFOS, human granulosa cells, reproductive toxicity, transcriptomics, PBTK, follicular fluid, human-equivalent dose, bioactivity exposure ratio, ART, occupational exposure

## Abstract

**STUDY QUESTION:**

Are bioactive human-equivalent doses (HEDs) of perfluorooctane sulfonate (PFOS), derived from long-term low-level *in vitro* exposure of human granulosa cells comparable to HEDs inferred from follicular fluid PFOS concentrations in women undergoing ART and in occupationally exposed women?

**SUMMARY ANSWER:**

The bioactive HEDs overlapped with and, in some cases, were lower than the median HEDs inferred from follicular fluid PFOS concentrations.

**WHAT IS KNOWN ALREADY:**

PFOS exposure is a growing public health concern, with evidence suggesting adverse female reproductive effects. However, the relevance of current human exposure levels to granulosa cell function remains unclear.

**STUDY DESIGN, SIZE, DURATION:**

Four independent vials of human granulosa cells (HGrC1 cells) were thawed and expanded into separate flasks (biological replicates). Cells were allocated to four experimental groups and exposed to PFOS (0.01, 0.1, or 1 µM) or vehicle control (0.05% DMSO) for up to 12 weeks, with re-dosing at each passage. Different apical endpoints, along with transcriptomic changes, were evaluated at designated time points. Clinical relevance of PFOS risk to human granulosa cells was assessed by integrating experimental data with physiologically based toxicokinetic (PBTK) modeling.

**PARTICIPANTS/MATERIALS, SETTING, METHODS:**

Viability of HGrC1 cells was assessed using the Alamar Blue assay. Estradiol and progesterone secretion were quantified by enzyme-linked immunosorbent assay. Flow cytometry was used to determine the proportions of live, apoptotic and necrotic cells, as well as cell cycle distribution. Global mRNA expression was assessed by DNA nanoball sequencing technology (DNBSEQ), whereas pathway-level molecular functions were derived using bioinformatic tools. Benchmark concentrations (BMCs) were calculated from key endpoints with concentration-dependent responses and used to estimate HEDs via PBTK modeling. These HEDs were compared with HEDs inferred from follicular fluid PFOS levels reported in the literature to derive bioactivity exposure ratios (BERs) and assess relevance to human exposure.

**MAIN RESULTS AND THE ROLE OF CHANCE:**

In HGrC1 cells, long-term PFOS exposure altered steroidogenesis, apoptosis/necrosis, cell cycle distribution (*P* < 0.05), and gene expression (at least 2-fold change, *Q*-value ≤ 0.05). Median transcriptomic HEDs were 18.1 (95% CI: 1.1–35.1) and 17.5 ng/kg bw/day (95% CI: 8–27.1) for 6- and 12-week exposures, respectively, with corresponding 5th percentile HEDs of 3.7 ng/kg bw/day (95% CI: 0.4–9.3) and 1.4 ng/kg bw/day (95% CI: 0.5–3.5). Pathway-level HEDs ranged from 2.8 to 24.1 ng/kg bw/day, with eicosanoid synthesis showing the greatest sensitivity. HEDs for apical endpoints ranged from 0.4 to 203 ng/kg bw/day, with the sub-G_1_ cell cycle phase being most sensitive. HEDs derived from the 5th percentile transcriptomic data, eicosanoid metabolism, and the sub-G_1_ phase yielded BERs below 1, indicating that PFOS levels measured in follicular fluid of ART patients may be sufficient to induce these biological effects. For occupational exposure, BERs derived from all endpoints were below 1. A subset of nine granulosa-cell genes, including *CYP1B1* and *TIPARP* (aryl hydrocarbon receptor signaling), showed HEDs that were below the follicular-fluid-inferred HED, highlighting potential high-priority targets and candidate biomarkers.

**LARGE SCALE DATA:**

Raw and processed RNA-sequencing data are deposited in NCBI Gene Expression Omnibus (GEO) under accession number GSE315651.

**LIMITATIONS, REASONS FOR CAUTION:**

The estimated exposure values were based on predictions from a PBTK model rather than empirical human exposure data. Also, differences in protein concentrations *in vitro* and *in vivo* may affect free PFOS levels and bioactivity estimates. We addressed this with additional adjustments for PFOS-albumin binding. Finally, follicular fluid PFOS concentrations in occupational settings were approximated from serum concentrations using blood-to-follicular fluid transfer efficiency (BFTE) values.

**WIDER IMPLICATIONS OF THE FINDINGS:**

Our findings suggest that PFOS concentrations in follicular fluid from women undergoing ART and those who have been occupationally exposed may be sufficient to perturb granulosa cell mRNA expression and key pathways, including eicosanoid, interleukin, and GPCR signaling. The identified genes may serve as candidate biomarkers linking PFOS exposure to clinical outcomes in ART settings. Overall, this study provides a framework for interpreting PFOS reproductive toxicity and refining health-protective exposure thresholds.

**STUDY FUNDING/COMPETING INTEREST(S):**

This research was supported by the Ministry of Science, Technological Development and Innovation of the Republic of Serbia (Faculty of Sciences, Novi Sad: Grants No. 451-03-137/2025-03/200125 & 451-03-136/2025-03/200125), the Institute of Physics, Belgrade, National Institute of the Republic of Serbia, and the Science Fund of the Republic of Serbia, Grant No. 7010, ‘Integration of Biological Responses and PBTK Modeling in Chemical Toxicity Assessment: А Case Study of Perfluorooctanoic Acid (PFOA)—ToxIN’. The authors declare no conflicts of interest.

WHAT DOES THIS MEAN FOR PATIENTS? Environmental pollutants are recognized as potential contributors to infertility. Perfluorooctane sulfonate (PFOS) is a man-made chemical that has been used in various industrial applications and consumer products. It does not break down easily in the environment and has been detected in the follicular fluid of women undergoing fertility treatment. Follicular fluid surrounds the egg in the ovary and is in direct contact with granulosa cells, which support egg development and produce reproductive hormones. This raises concerns about the possible effects of PFOS exposure on fertility.

In this study, we exposed human granulosa cells over several weeks to low levels of PFOS, at doses similar to those measured in follicular fluid. Exposure changed hormone production, cell cycle regulation, and gene activity in these cells. Using a computer model, we estimated PFOS intake that could lead to ovarian concentrations similar to those affecting granulosa cells in our experiments. The results suggest that PFOS levels reported in follicular fluid, particularly in highly exposed women, may affect granulosa cell function and molecular pathways related to ovulation and inflammation. The study also identifies candidate cellular biomarkers and pathways that may explain how PFOS exposure could influence egg quality and fertility treatment outcomes. Since this research was conducted on isolated cells, it cannot capture all the complex interactions in the ovary, and further studies are necessary to confirm these effects in women.

## Introduction

Perfluorooctane sulfonate (PFOS) is a synthetic fluorinated compound belonging to the per- and poly-fluoroalkyl substances (PFAS) family. Developed in the late 1940s, PFOS gained extensive industrial and commercial use due to its exceptional chemical stability, surfactant properties, and resistance to degradation. However, its extreme environmental persistence, strong bioaccumulative potential, and capacity for long-range transport have made PFOS a global environmental concern, exemplifying the ongoing challenge of advancing industrial development while ensuring environmental protection and human well-being. Mounting evidence of PFOS toxicity has prompted intensified regulatory attention, leading to its phase-out by major manufacturers and classification as a persistent organic pollutant. Nevertheless, PFOS continues to be produced in certain regions for limited essential applications and remains detectable worldwide in water, soil, wildlife and human serum and tissues (Suman and Kwak, 2025).

Across studies summarized by Zhang *et al.* (2025), serum PFOS concentrations among women of reproductive age ranged from 0.2 to 187.2 ng/ml. Substantially higher levels have been reported among occupationally exposed individuals. Women employed at a fluorochemical manufacturing facility exhibited median serum concentrations of 1,155 ng/ml (range: 460–25,000 ng/ml) in 2008 and 1,231 ng/ml (range: 426–19,564 ng/ml) in 2012, reflecting the compound’s persistence in the human body (Fu *et al.*, 2016). PFOS can also cross the blood–follicle barrier and accumulate in follicular fluid, with levels strongly correlated with those in serum (Heffernan *et al.*, 2018). Although follicular fluid concentrations have not been assessed in occupational settings, studies of women undergoing ART report PFOS levels ranging from 0.1 to 181 ng/ml (Bellavia *et al.*, 2023; Zeng *et al.*, 2023; Clark *et al.*, 2024; Li *et al.*, 2024; Bian *et al.*, 2025; Zhang *et al.*, 2025; Boney *et al.*, 2026).

These exposure profiles raise substantial concern regarding the potential impact of PFOS on female reproductive health. Epidemiological studies have linked PFOS exposure to reduced fecundability (Fei *et al.*, 2009; Cohen *et al.*, 2023), increased infertility risk (Fei *et al.*, 2009), menstrual cycle irregularities (Zhou *et al.*, 2017; Heffernan *et al.*, 2018), higher prevalence of polycystic ovary syndrome (Heffernan *et al.*, 2018; Zhang *et al.*, 2024), premature ovarian insufficiency (Zhang *et al.*, 2018), and endometriosis (Campbell *et al.*, 2016). Experimental studies in rodents further support these associations, demonstrating that chronic PFOS exposure disrupts estrous cyclicity, reduces the number of mature follicles and corpora lutea, and increases follicular atresia (Feng *et al.*, 2015). Collectively, these findings suggest that the ovarian follicle represents a target of PFOS toxicity.

To better understand the reproductive effects of PFOS, it is essential to examine its impact on granulosa cells, the functional core of the ovarian follicle (Cavalcanti *et al.*, 2023). *In vitro* studies indicate that PFOS can disrupt granulosa-cell function, although reported effects vary by species, cell model, and exposure conditions. PFOS did not alter estradiol or progesterone production in porcine granulosa cells (Chaparro-Ortega *et al.*, 2018). In the human granulosa cell line KGN, 48-h exposure reduced cell viability and induced apoptosis and autophagy (Gao *et al.*, 2024), while 72-h exposure stimulated proliferation in KGN and COV434 cells (Gogola *et al.*, 2019). Given continuous human exposure through drinking water, diet and consumer products, and the long biological half-life of PFOS in serum (∼5.4 years) (Suman and Kwak, 2025), chronic low-dose accumulation represents the most realistic exposure scenario for risk assessment. However, existing studies address short-term exposure, leaving critical gaps in understanding direct PFOS effects in human granulosa cells under environmentally relevant conditions.

As new PFOS remediation technologies emerge, strengthening regulatory decision-making remains essential for protecting human health and the environment. However, substantial uncertainty challenges PFOS risk assessment, as reflected in the wide range of proposed reference doses (RfDs), spanning a 600-fold difference from 0.1 ng/kg body weight (bw)/day (United States Environmental Protection Agency (USEPA), 2024) to 60 ng/kg bw/day (Health Canada, 2018). The European Food Safety Authority (EFSA) set a tolerable daily intake of 1.86 ng/kg bw/day in 2018 for PFOS exposure (EFSA Panel on Contaminants in the Food Chain (CONTAM) *et al.*, 2018), later revising it to 0.63 ng/kg bw/day in 2020, for combined exposures to perfluorooctanoic acid (PFOA), perfluorononanoic acid, perfluorohexanesulfonic acid, and PFOS (EFSA Panel on Contaminants in the Food Chain (EFSA CONTAM Panel) *et al.*, 2020). A recent international expert review under the Alliance for Risk Assessment re-evaluated the available evidence and concluded that epidemiological data are too confounded to support reliable safe-dose estimation, while animal bioassays and differing uncertainty factors largely explain discrepancies among RfDs. Using benchmark dose modeling based on rat and nonhuman primate studies, the review proposed a narrower safe-dose range of 20–100 ng/kg bw/day (Dourson *et al.*, 2026). Nevertheless, uncertainty remains regarding how well animal models capture human-specific biological responses, underscoring the need for human-relevant *in vitro* data to support PFOS risk evaluation.

To address these gaps, the present study investigated the effects of PFOS in the non-luteinized human granulosa cell line, HGrC1 following long-term exposure (6 and 12 weeks) at concentrations comparable to those detected in human serum and follicular fluid. After quantifying changes in key cellular and functional endpoints, we derived benchmark concentrations (BMCs) and applied physiologically based toxicokinetic (PBTK) modeling to translate *in vitro* BMCs from transcriptomic and apical endpoints into human-equivalent doses (HEDs). These HEDs were compared with population exposure estimates to generate bioactivity exposure ratios (BERs), thereby clarifying whether PFOS-induced granulosa-cell effects occur within the range of real-world human exposure.

## Materials and methods

### Chemicals

Dulbecco’s Modified Eagle Medium/Nutrient Mixture F-12 (DMEM/F-12) and HEPES were obtained from Gibco™, Thermo Fisher Scientific (Waltham, MA, USA). Penicillin-streptomycin solution, L-glutamine, trypsin-EDTA solution, dimethyl sulfoxide (DMSO), testosterone, and Triton™ X-100 were purchased from Sigma-Aldrich, Merck KGaA (Darmstadt, Germany). Fetal bovine serum (FBS) was obtained from Capricorn Scientific (Ebsdorfergrund, Germany), while PFOS (potassium salt, ≥ 98% purity, CAS No. 2795-39-3) was purchased from Supelco, Merck KGaA. The alamarBlue™ Cell Viability Reagent, propidium iodide (PI), and TRIzol™ reagent were purchased from Invitrogen, Thermo Fisher Scientific. Estradiol and progesterone ELISA kits were purchased from Cayman Chemical (Ann Arbor, MI, USA), while Pierce™ BCA Protein Assay Kit and RNase A were obtained from Thermo Fisher Scientific. Annexin V-FITC reagent was obtained from Elabscience (Houston, TX, USA). All other chemicals were of analytical grade.

### Culture of HGrC1 cells

The non-luteinized human granulosa cell line HGrC1 (Bayasula *et al.*, 2012) was kindly provided by Dr Akira Iwase (Nagoya University, Japan). Cells were maintained in DMEM/F-12 supplemented with 100,000 U/l penicillin, 100 mg/l streptomycin, 1.4 g/l sodium bicarbonate, 2 mM L-glutamine, and 10% FBS (complete culture medium; pH 7.4), at 37 °C in a humidified atmosphere containing 5% CO_2_. Subculturing was performed twice weekly, using 0.25% trypsin–EDTA for detachment (3 min, 37 °C).

### Long-term exposure to PFOS

Four vials of HGrC1 cells cryopreserved on different dates (biological replicates) were thawed and cultured separately. After a 2-week acclimation period, cells were subcultured into four 25 cm^2^ flasks per replicate and exposed to PFOS (0.01, 0.1, or 1 µM), or vehicle control (0.05% DMSO) for 12 weeks. Selected PFOS concentrations are within the range reported in human follicular fluid and serum, spanning from 0.0002 µM (Petro *et al.*, 2014) to 49.99 µM (Fu *et al.*, 2016). Cells were subcultured twice weekly and reseeded in complete culture medium supplemented with 100 nM testosterone to serve as an aromatase substrate for estradiol production. Freshly prepared PFOS or vehicle were added 3 h after reseeding, to avoid interference with cell attachment. The following apical endpoints were analyzed: cell viability after 3, 6, 9, and 12 weeks of exposure, as well as hormone secretion, apoptosis/necrosis, and cell cycle distribution after 6 and 12 weeks. Transcriptomic changes were evaluated after 6 and 12 weeks of PFOS exposure.

### Alamar blue assay

Cell viability was assessed using the alamarBlue™ reagent. One passage before the end of each exposure period, cells were seeded into 96-well plates (2 × 10^4^ cells/well/200 µl) and exposed to PFOS or vehicle. After exposure, cells were incubated with 60 µl of 10% alamarBlue™ solution (2.5 h, at 37 °C in the dark under 5% CO_2_). Blank wells containing only the alamarBlue™ solution were included to correct for background fluorescence. Fluorescence was measured at 540 nm excitation and 590 nm emission using a Fluoroskan Ascent microplate reader (Thermo LabSystems Inc., Thermo Fisher Scientific). Each experimental condition was assessed using six technical replicates.

### ELISA

Estradiol and progesterone concentrations in conditioned media collected from each flask after exposure were quantified using commercial ELISA kits, following the manufacturer’s instructions. Each condition was analyzed in duplicate and normalized to total cellular protein content (pg hormone/mg protein). Total protein levels in corresponding lysates of the cells collected from culture flasks were measured using the BCA assay. Absorbances for hormone and protein measurements were recorded at 405 and 540 nm, respectively, using a Multiskan EX microplate reader (Thermo LabSystems Inc., Thermo Fisher Scientific).

### Apoptosis assay

For apoptosis assessment, cells were harvested from flasks after exposure, together with the corresponding growth medium (containing already detached cells). A total of 2 × 10^6^ cells were incubated for 20 min with 70 µl of staining solution (3.18 µl Annexin V-FITC and 3.18 µl PI [100 µg/ml] in Annexin V Binding Buffer [10 mM HEPES, 140 mM NaCl, and 2.5 mM CaCl_2_; pH 7.4]). Subsequently, an additional 70 µl of Annexin V Binding Buffer was added. Samples were filtered through nylon meshes and analyzed using an Amnis^®^ ImageStream^®^ X Mk II Imaging Flow Cytometer (Luminex Corporation, Austin, TX, USA). For each sample, 15,000 properly focused single cells were acquired using a 488-nm excitation laser operated at 20 mW for both FITC and PI channels. Data were processed using IDEAS^®^ 6.2 software (Luminex Corporation) and expressed as percentages of live, apoptotic (early and late), and necrotic cells.

### Cell cycle analysis

For cell cycle analysis, cells were harvested from flasks after exposure, together with the corresponding growth medium (containing already detached cells). A total of 2 × 10^6^ cells were resuspended in ice-cold phosphate-buffered saline (PBS) and added dropwise to 70% ice-cold ethanol under constant gentle agitation. After fixation at 4 °C, cells were washed with ice-cold PBS and incubated for 20 min in 150 µl of PI staining solution (10 µg/ml PI, 0.1% Triton X-100, and 100 µg/ml RNase A in PBS). Samples were filtered through nylon meshes and analyzed using the Amnis^®^ ImageStream^®^ X Mk II Imaging Flow Cytometer (Luminex Corporation). For each sample, 15,000 properly focused single cells were acquired using a 488 nm excitation laser, operated at 5 mW for the PI channel. Data were processed using IDEAS^®^ 6.2 software (Luminex Corporation) and reported as percentages of cells in sub-G_1_, G_0_/G_1_, S, and G_2_/M stages of the cell cycle.

### RNA extraction

After exposure, 1 × 10^6^ cells were collected from each flask for RNA extraction using TRIzol™ reagent (0.5 ml), according to the manufacturer’s protocol. Concentration and purity of isolated RNA were assessed using a BioSpec-nano spectrophotometer (Shimadzu Corporation, Kyoto, Japan).

### RNA sequencing

RNA was sequenced by BGI Tech Solutions (Warsaw, Poland) using the DNBSEQ™ platform. RNA quantity and integrity were evaluated using the 4150 TapeStation System (Agilent Technologies, Santa Clara, CA, USA), and all samples met library preparation criteria. After sequencing, raw reads were filtered to remove adaptor sequences, reads with > 1% ambiguous bases, and low-quality reads (> 40% bases with *Q* ≤ 20). Clean reads were aligned to the *Homo sapiens* reference genome (GRCh38.p14) using HISAT (Kim *et al.*, 2015) or to reference genes using Bowtie2 (Langmead and Salzberg, 2012). Gene expression was quantified via RSEM (Li and Dewey, 2011) and differential expression analysis was conducted using DESeq2 (Love *et al.*, 2014). Genes with an adjusted *P*-value (calculated using a false discovery rate-based approach, *Q*-value) ≤ 0.05 and an absolute log2-fold change (|log2FC|) ≥ 1 were considered differentially expressed (DEGs). Downstream functional annotation of hierarchical DEG expression clusters is described in the Supplementary Materials and Methods.

### BMC modeling

BMC modeling was performed on apical endpoints and log2-transformed DESeq2-normalized read counts using BMDExpress3 (Yang *et al.*, 2007). Briefly, probes exhibiting monotonic concentration-response patterns were identified using the Williams trend test (500 permutations, 1.5-fold change, *P *< 0.05). Selected probes were modeled using EPA Benchmark Dose Software (BMDS, U.S. Environmental Protection Agency, Washington, DC, USA) models (Linear, Exp3, Exp5, Poly2, Poly3, and Hill) with a benchmark response of 1 standard deviation (SD). The best-fitting model was selected based on the Nested chi-square test (*P*-value cutoff: 0.05). The software calculated the BMC, and its lower and upper confidence limits (BMCL and BMCU, respectively). Results were subsequently filtered to retain reliable BMCs, defined as those not exceeding the tested concentration range and with BMCU/BMCL < 40.

PFOS binds strongly to plasma proteins, primarily albumin, leaving only a small free fraction (predicted to be ∼0.33%) available for biological activity (Argoul *et al.*, 2025). In contrast, the free fraction in the *in vitro* exposure system containing 10% FBS is higher due to lower protein content. Consequently, exposure concentrations producing effects *in vitro* may not affect granulosa cells in real life, potentially leading to inaccurate risk assessment. To account for differences between *in vitro* and *in vivo* conditions, obtained BMCs were adjusted for albumin binding as described in the Supplementary Materials and Methods and these corrected values were included in downstream HED and BER calculations.

### Pathway enrichment analysis

Reactome pathway enrichment analysis was performed using Enrichr (Chen *et al.*, 2013; Kuleshov *et al.*, 2016; Xie *et al.*, 2021) on genes with determined BMC values following 6- and 12-week exposures of HGrC1 cells to PFOS. The analysis was conducted for all genes collectively, as well as for gene subsets stratified according to the direction of expression change.

### PBTK modeling

**Figure 1.**
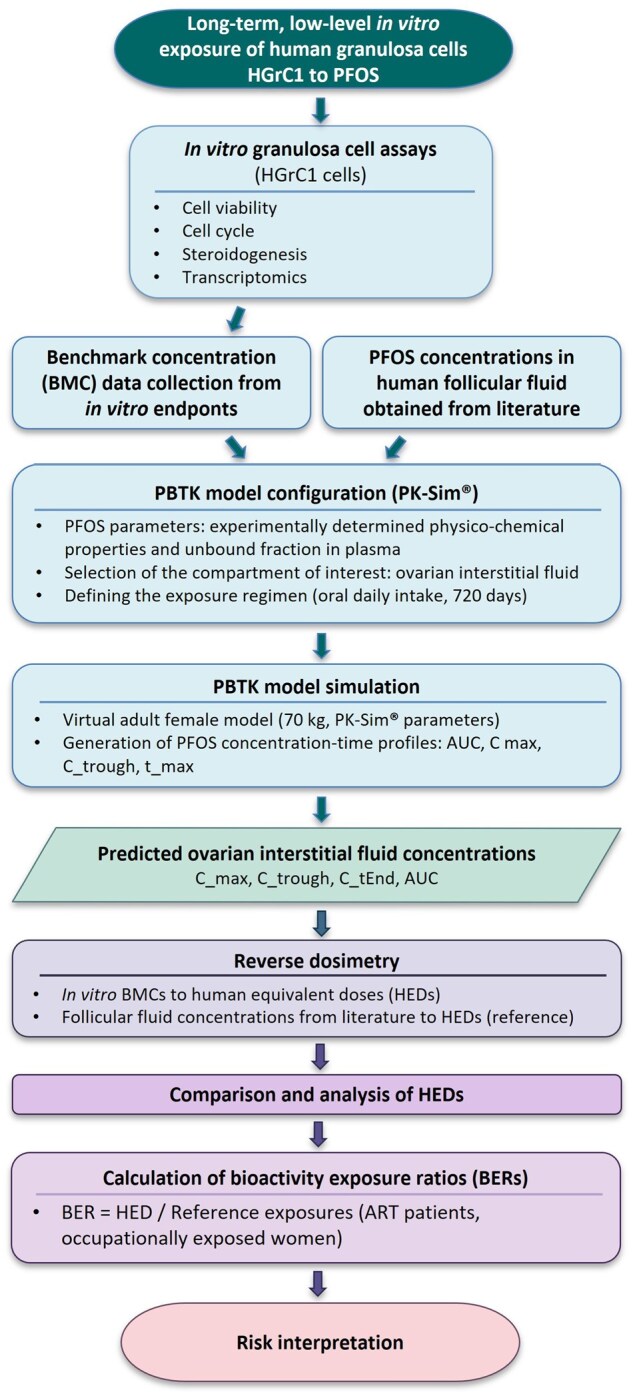
**Schematic overview of the workflow used to translate *in vitro* bioactivity data obtained in human granulosa cells (HGrC1) to human exposure estimates using physiologically based toxicokinetic modeling**. Physiologically based toxicokinetic (PBTK) model for PFOS was developed using PK-Sim^®^. C_max, maximum concentration; C_trough, trough concentration; t_max, time to maximum concentration; C_tEnd, concentration at the end of simulation; BER, bioactivity exposure ratio; HED, human-equivalent dose.

PBTK model for PFOS was developed using PK-Sim^®^ (Open Systems Pharmacology Suite) (Willmann *et al.*, 2003) to predict chronic PFOS distribution, with a specific focus on the ovaries (gonads). PFOS concentrations reported in human follicular fluid and *in vitro* BMCs, both representing total PFOS (bound and unbound), were used as target internal concentrations. Accordingly, ovarian interstitial fluid (total concentration) was selected as the compartment of interest, as it represents the closest physiological surrogate for both follicular fluid and cell culture medium. Experimentally determined physicochemical properties of PFOS were incorporated into PK-Sim, using the ‘Compound Properties’ module. These included the free fraction in plasma, molecular weight, aqueous solubility, number of halogen atoms, pKa, and solubility at physiological pH relevant to ovarian tissue, enabling accurate representation of PFOS ionization state, protein binding, and tissue distribution within the simulated biological system. The plasma free fraction was set to 0.33% based on reported human data (Argoul *et al.*, 2025). Exposure was defined as daily oral intake over two years (720 days), with doses adjusted to reproduce PFOS plasma concentrations observed in human biomonitoring studies. Simulations were performed for an adult female virtual individual (default PK-Sim anatomical parameters, 70 kg), to reflect conditions relevant for distribution within the female reproductive system. The model computed detailed concentration–time profiles for ovarian interstitial fluid, allowing characterization of PFOS bioaccumulation dynamics. PK-Sim outputs included area under the concentration–time curve (AUC_tD1–tD2, AUC_tEnd, and normalized values), maximum concentration (C_max), trough concentration (C_trough), concentration at the end of simulation (C_tEnd), and time to maximum concentration (t_max) for both early exposure and steady-state conditions (Fig. 1). The model output is described in the Supplementary Materials and Methods.

### BER calculation

PFOS levels detected in follicular fluid of ART patients (summarized in Supplementary Table S1) were used as target concentrations in ovarian interstitial fluid to derive HEDs. HEDs corresponding to the average of median concentrations across studies (3.5 ng/kg bw/day), as well as the minimum (0.1 ng/kg bw/day) and maximum (172 ng/kg bw/day) values, were used as reference exposures for BER calculation to define a range capturing variability in human exposure. To account for the risks associated with occupational exposure, additional reference HEDs (median, min–max: 877.3, 303.6–13942.7 ng/kg bw/day) were included. Because follicular fluid PFOS levels under occupational exposure have not been reported, they were predicted from serum concentrations in exposed women (Fu *et al.*, 2016) using the reported blood-to-follicular fluid transfer efficiency (BFTE) of 0.75 (Zhang *et al.*, 2025). These estimated follicular fluid concentrations were calculated using the equation *C*_follicular fluid_ = *C*_serum_ × BFTE and are provided in Supplementary Table S1. BERs were calculated by dividing median transcriptomic, pathway-level, and apical HEDs with reference exposures. Additionally, for transcriptomic data, BERs were also calculated by dividing 5th percentile HEDs with reference exposures.

### Statistical analysis

Statistical analyses were performed using GraphPad Prism 8 (GraphPad Software, San Diego, CA, USA) and one-way ANOVA followed by Dunnett’s *post hoc* test for multiple comparisons.

## Results

### Apical endpoints in HGrC1 cells following long-term low-level PFOS exposure

#### Cellular viability

**Figure 2.**
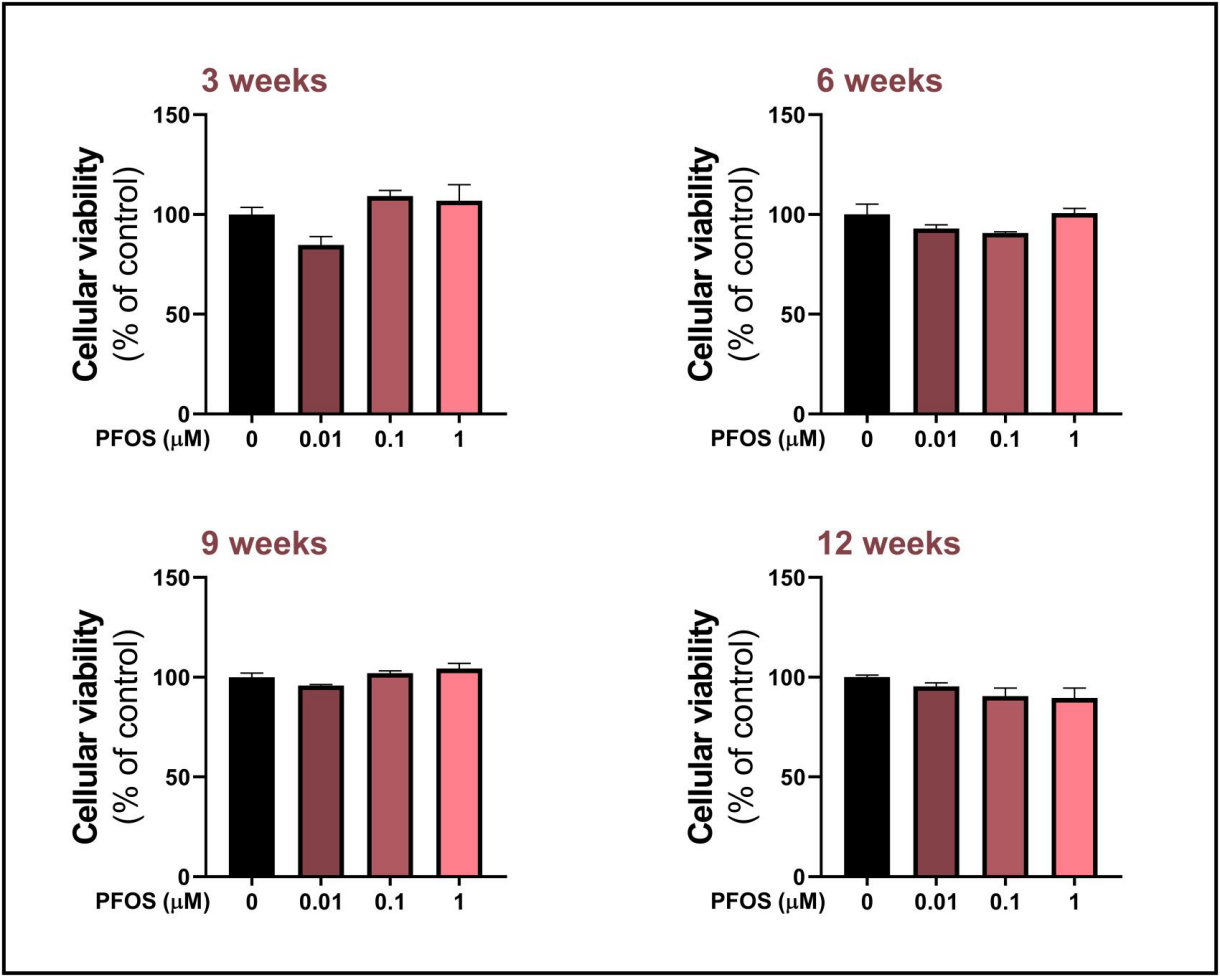
**Viability of human granulosa cells (HGrC1) after long-term, low-level perfluorooctane sulfonate (PFOS) exposure**. Cells were exposed to 0.01, 0.1, or 1 µM PFOS, or to vehicle control (0 µM PFOS) for 3, 6, 9, and 12 weeks. Viability was assessed using the Alamar Blue assay and expressed relative to control (set as 100%). Results are presented as the mean ± standard error of the mean (SEM), from four biological replicates. Data were analyzed using one-way ANOVA followed by Dunnett’s *post hoc* test for multiple comparisons.

Alamar Blue assay revealed no statistically significant changes in the viability of HGrC1 cells after 3, 6, 9, or 12 weeks of exposure to PFOS (Fig. 2).

#### Estradiol and progesterone secretion

**Figure 3.**
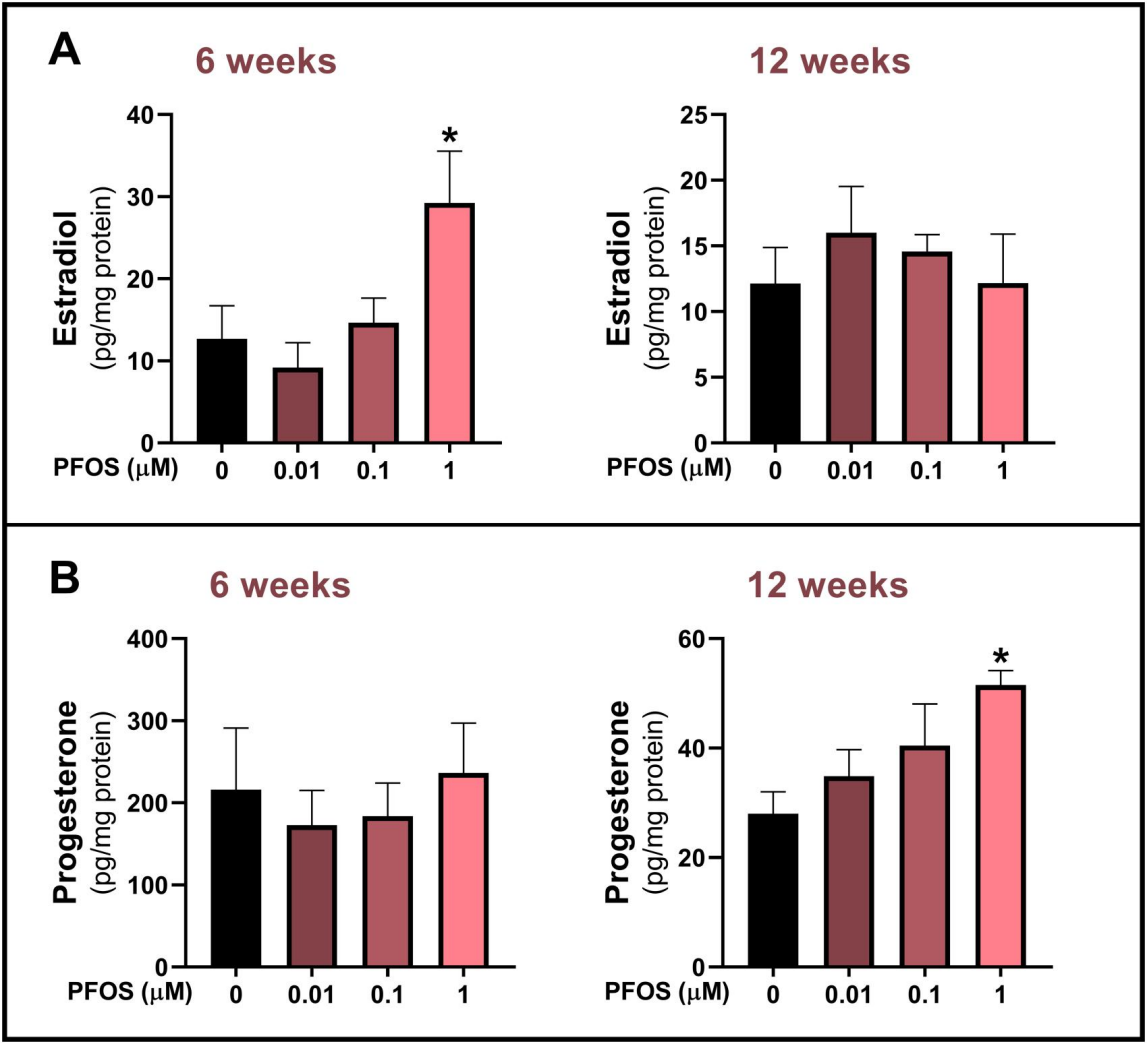
**Estradiol and progesterone levels in the culture medium of human granulosa cells (HGrC1) after long-term, low-level perfluorooctane sulfonate (PFOS) exposure**. Cells were exposed to 0.01, 0.1, or 1 µM PFOS, or to vehicle control (0 µM PFOS) for 6 and 12 weeks. (**A**) Estradiol and (**B**) progesterone levels in conditioned media were quantified using ELISA and expressed as pg per mg of total cellular proteins, determined via BCA assay. Results are presented as the mean ± SEM, from four biological replicates. Data were analyzed using one-way ANOVA followed by Dunnett’s *post hoc* test for multiple comparisons. *P < 0.05 vs control.

Next, we evaluated the levels of estradiol and progesterone in the culture medium collected from HGrC1 cells following long-term, low-level PFOS exposure. Estradiol was significantly elevated after 6 weeks of exposure to 1 µM PFOS, with no statistically significant changes after 12 weeks (Fig. 3A), when compared with no exposure. In contrast, progesterone remained unchanged after 6 weeks but was increased following 12 weeks of exposure to 1 µM PFOS, when compared with no exposure. Interestingly, progesterone levels in the control group were markedly lower after 12 weeks than after the 6-week exposure period (Fig. 3B).

#### Apoptosis and necrosis

**Figure 4.**
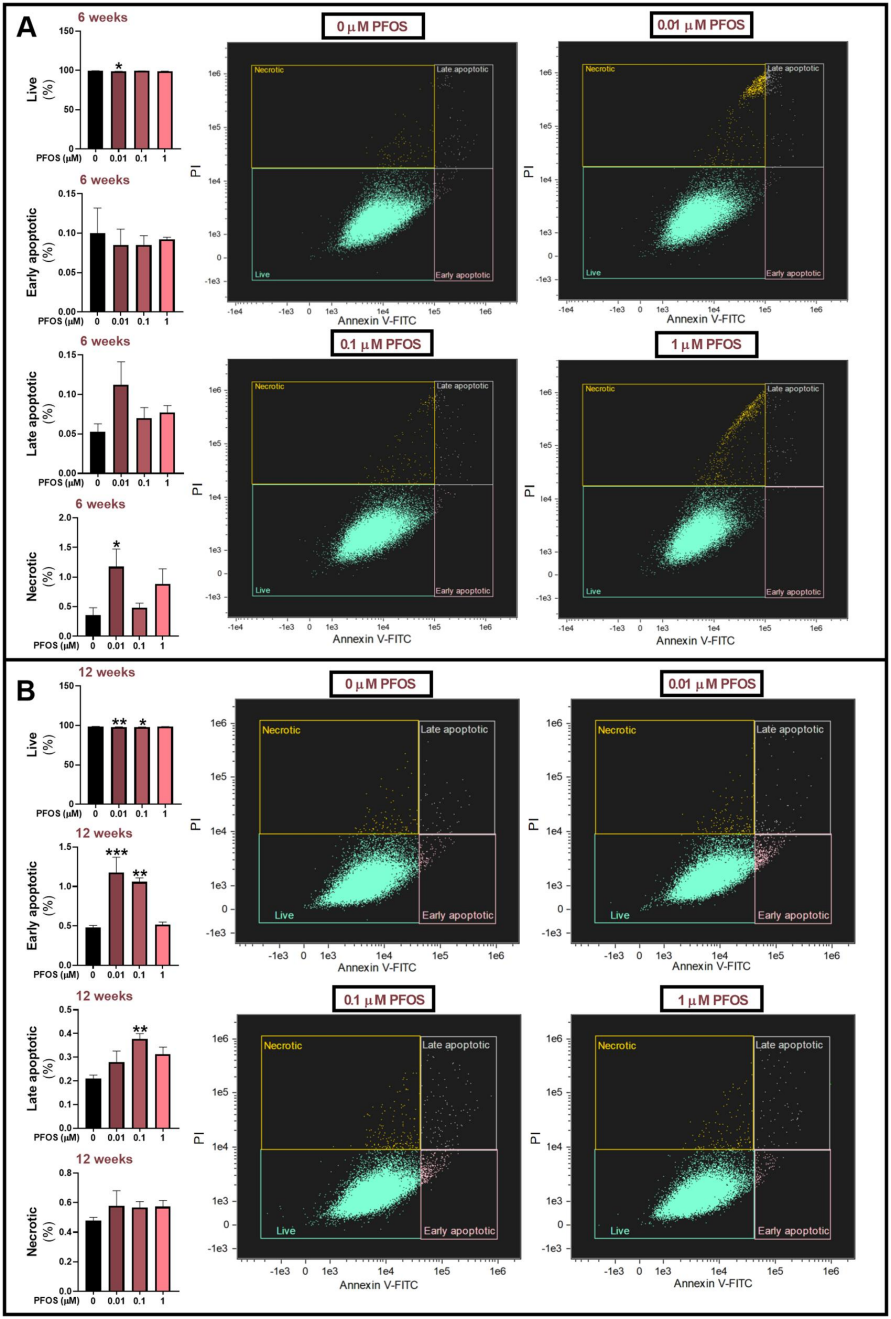
**The effects of long-term, low-level exposure of human granulosa cells (HGrC1) to perfluorooctane sulfonate (PFOS) on apoptosis and necrosis**. Cells were exposed to 0.01, 0.1, or 1 µM PFOS, or to vehicle control (0 µM PFOS). Flow cytometry with Annexin V-FITC/PI dual staining was used to assess cell viability after (**A**) 6 and (**B**) 12 weeks of exposure. Live (Annexin V-FITC^−^/PI^−^), early apoptotic (Annexin V-FITC^+^/PI^−^), late apoptotic (Annexin V-FITC^+^/PI^+^), and necrotic (Annexin V-FITC^−^/PI^+^) cells are presented as % of the total population of well-focused, single cells. Results are presented as the mean ± SEM, from four biological replicates. Data were analyzed using one-way ANOVA followed by Dunnett’s *post hoc* test for multiple comparisons. *P < 0.05 vs control; **P < 0.01 vs control; ***P < 0.001 vs control.

To further investigate the effects of PFOS, we examined whether long-term exposure induced apoptosis or necrosis in HGrC1 cells. The results showed that 0.01 µM PFOS significantly decreased the percentage of live cells while increasing the percentage of necrotic cells after a 6-week exposure (Fig. 4A). After 12 weeks, both 0.01 and 0.1 µM PFOS significantly decreased the percentage of live cells while increasing the percentage of early apoptotic cells. Furthermore, the percentage of late apoptotic cells increased after 12-week exposure to 0.1 µM PFOS (Fig. 4B). Despite these changes, most cells remained viable after 6- and 12-week exposures, and the overall proportion of dying cells in the total population was low.

#### Cell cycle distribution

**Figure 5.**
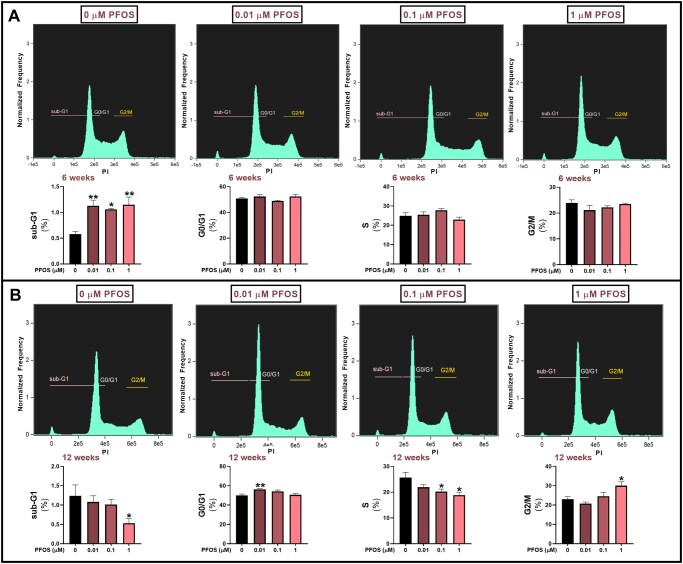
**The effects of long-term, low-level exposure of human granulosa cells (HGrC1) to perfluorooctane sulfonate (PFOS) on cell cycle distribution**. Cells were exposed to 0.01, 0.1, or 1 µM PFOS, or to vehicle control (0 µM PFOS), stained with PI, and analyzed by flow cytometry after (**A**) 6 and (**B**) 12 weeks. Cells at each stage of the cell cycle are presented as % of the total population of well-focused, single cells. Results are presented as the mean ± SEM, from four biological replicates. Data were analyzed using one-way ANOVA followed by Dunnett’s *post hoc* test for multiple comparisons. *P < 0.05 vs control; **P < 0.01 vs control.

The results on cell cycle distribution showed that all tested concentrations of PFOS increased the sub-G_1_ fraction after 6 weeks of exposure (Fig. 5A). In contrast, the 12-week exposure to 1 µM PFOS decreased the sub-G_1_ and increased the G_2_/M fraction. Moreover, 0.01 µM PFOS increased the G_0_/G_1_ population, whereas 0.1 and 1 µM PFOS reduced the S phase fraction (Fig. 5B).

### Transcriptomic changes in HGrC1 cells following long-term, low-level exposure to PFOS

**Figure 6.**
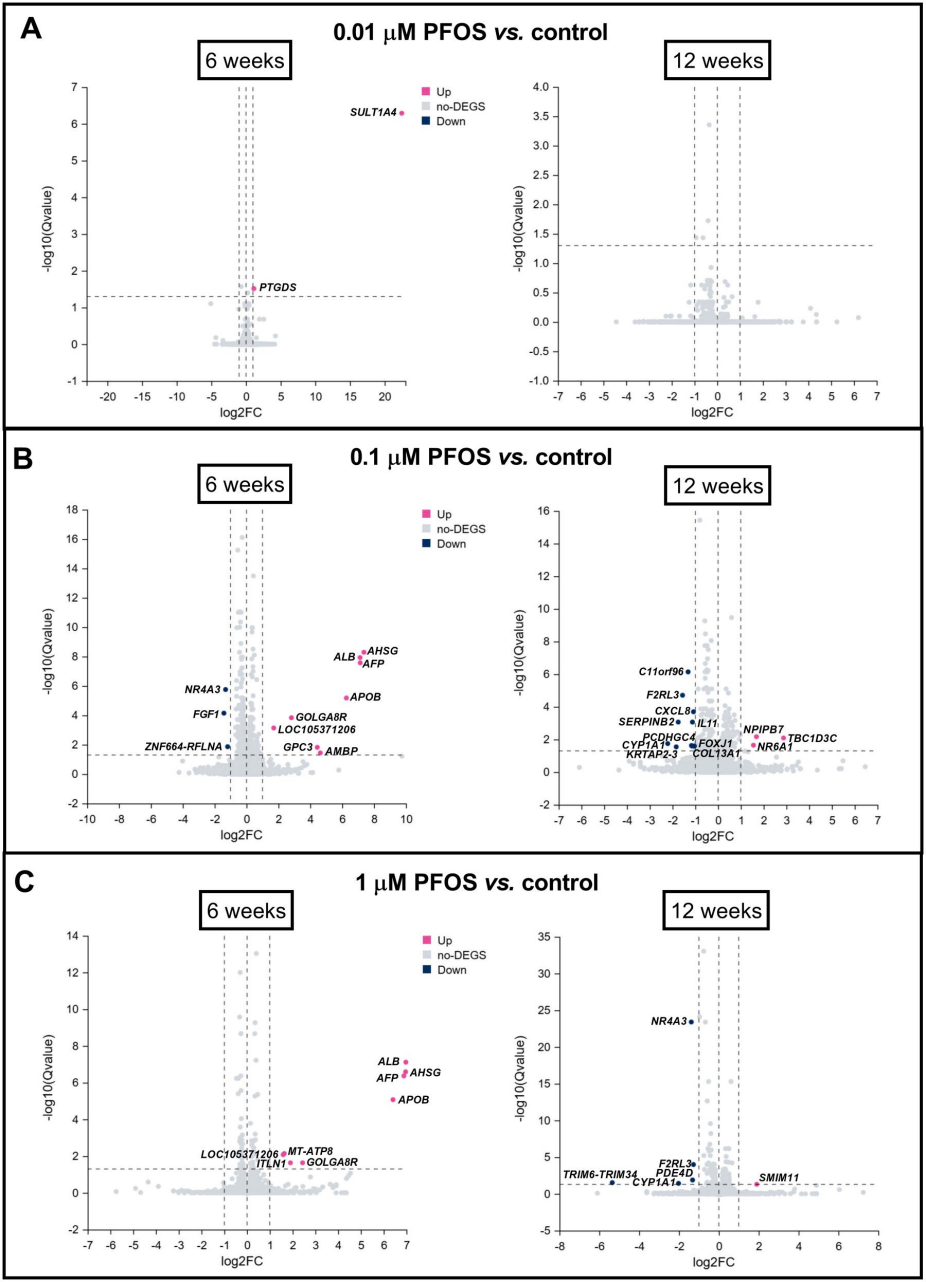
**Volcano plots of differentially expressed genes identified in human granulosa cells (HGrC1) following long-term, low-level perfluorooctane sulfonate (PFOS) exposure**. RNA sequencing was performed after 6 and 12 weeks of exposure to (**A**) 0.01, (**B**) 0.1, or (**C**) 1 µM PFOS, or to vehicle control. Differential expression analysis was performed using DESeq2. *X*-axis: log2-fold change (log2FC); *Y*-axis: −log10 transformed *Q*-value. Genes with *Q*-value ≤ 0.05 and |log2FC| ≥ 1 were considered differentially expressed (DEGs). Significantly upregulated genes are presented as pink dots, downregulated genes are blue dots, and non-significant changes are shown as grey dots.

Transcriptome analysis revealed DEGs (at least 2-fold change, *Q*-value ≤ 0.05) in HGrC1 cells after 6- and 12-week exposures to PFOS. At 0.01 µM PFOS, minimal changes were observed after 6 weeks and none after 12 weeks (Fig. 6A), while 0.1 and 1 µM PFOS elicited more pronounced transcriptional responses, with a predominance of upregulated genes at 6 weeks and downregulated genes at 12 weeks (Fig. 6B and C, Supplementary Fig. S1A). A Venn diagram revealed that no DEGs were shared across all three exposure groups. However, several genes were shared between the 0.1 and 1 µM groups at each time point, including *AHSG*, *ALB*, *AFP*, *APOB*, *GOLGA8R*, *LOC105371206* at 6 weeks, and *F2RL3* and *CYP1A1* at 12 weeks (Supplementary Fig. S1B).

Hierarchical clustering of DEGs based on their expression profiles identified four distinct clusters (Supplementary Fig. S1A). Functional annotation of differential expression clusters is presented in Supplementary Fig. S1C. At 6 weeks, cluster 1 (exhibiting marked upregulation at 0.01 µM PFOS with a trend of progressive decline at higher concentrations) was associated with GO terms related to xenobiotic, lipid, and catecholamine metabolism and cellular responses to dopamine. Cluster 2, which showed pronounced trend of upregulation at 0.1 and 1 µM PFOS, was most strongly associated with signal transduction, negative regulation of cell proliferation, protein catabolism, sexual reproduction, post-embryonic development, oxidant detoxification, and phagocytosis. Cluster 3, downregulated at 0.1 µM PFOS, was associated with epithelial cell proliferation, transcriptional regulation, cell migration, division, differentiation, and wound healing. Cluster 4, characterized by weak or minimal upregulation, was associated with ion transport, lipid metabolism, and Golgi organization. At 12 weeks, cluster 1 showed a trend of upregulation, most prominently at 0.1 µM, and was related to gamete generation and transcriptional regulation by RNA polymerase II. Cluster 2 displayed strong downregulation at 1 µM and was not associated with any GO term. Cluster 3 was strongly downregulated at higher concentrations and was linked to steroid, fatty acid, and xenobiotic metabolism, as well as the positive regulation of the G_1_/S cell cycle transition. Cluster 4 exhibited moderate downregulation and was associated with signal transduction, DNA-templated transcription, cell differentiation, cell adhesion, and negative regulation of G-protein-coupled receptor signaling.

### BMC estimates across transcriptomic and apical data in HGrC1 cells following long-term low-level exposure to PFOS

**Figure 7.**
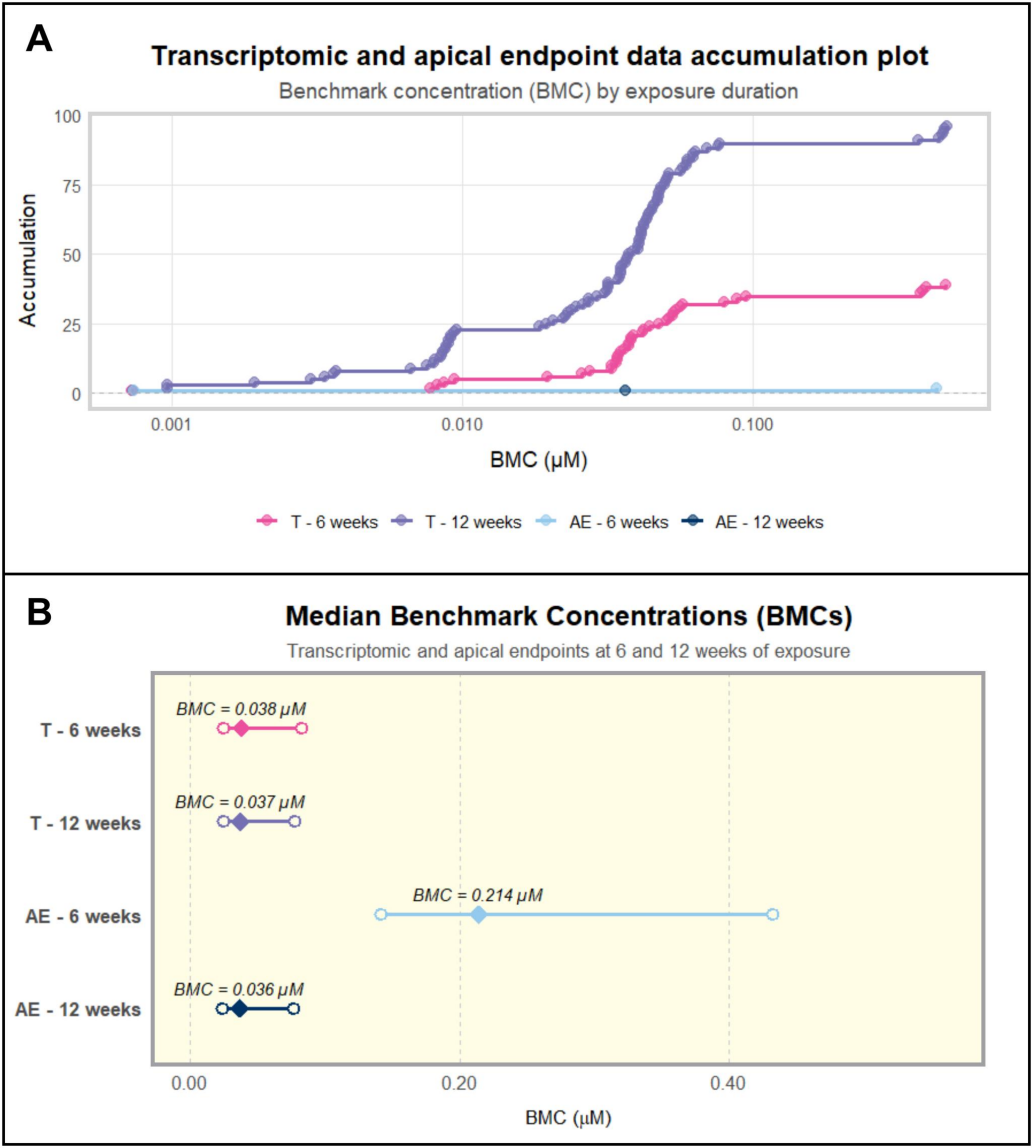
**Estimated benchmark concentrations in human granulosa cells (HGrC1) following long-term, low-level exposure to perfluorooctane sulfonate (PFOS)**. Probes with concentration–response relationships were identified using the Williams trend test and the best-fitting models were selected based on the Nested chi-square test. (**A**) Accumulation plot of estimated benchmark concentrations (BMC) for transcriptomic data (T) and apical endpoints (AE) following 6- and 12-week PFOS exposures. (**B**) Range plot illustrating median BMC (diamond) and benchmark concentration lower (BMCL) and upper (BMCU) confidence limits (circles) for transcriptomic (T) and apical (AE) data, following 6- and 12-week PFOS exposures.

Estimated BMC values for transcriptomic responses at both 6- and 12-week exposures reached the dynamic response phase between 0.01 and 0.1 µM PFOS. Only two apical endpoints at 6 weeks (estradiol secretion and sub-G_1_ stage of the cell cycle) and one apical endpoint at 12 weeks (fraction of late apoptotic cells) exhibited a concentration-dependent response (Fig. 7A). Median BMCs were similar for 6- and 12-week transcriptomic and 12-week apical endpoint data and ranged from 36.5 nM (BMCL–BMCU: 23.9–76 nM) to 38.1 nM (BMCL–BMCU: 24.7–82.5). At 6 weeks, apical endpoints had a higher median BMC of 214 nM (BMCL–BMCU: 141.3–432.4 nM) (Fig. 7B). BMC values corrected for albumin binding are presented in Supplementary Fig. S2.

### Pathway enrichment analysis

**Figure 8.**
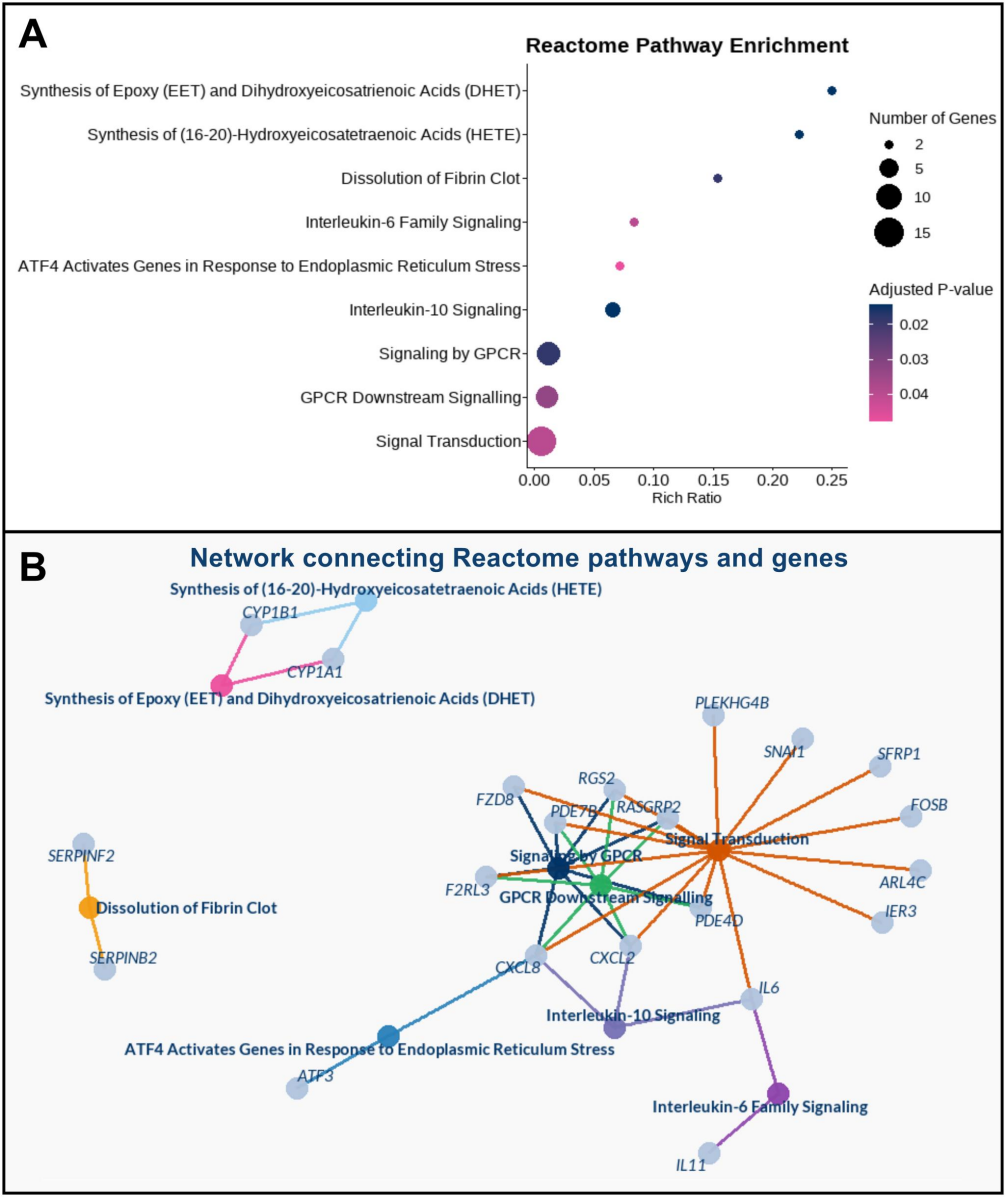
**Pathways significantly annotated with genes exhibiting a trend of decreased expression in human granulosa cells (HGrC1) following 12 weeks of perfluorooctane sulfonate (PFOS) exposure**. (**A**) Dotplot illustrating the results of pathway enrichment analysis, showing significantly enriched Reactome pathways identified using Enrichr. (**B**) Gene interaction networks corresponding to each enriched pathway.

We performed pathway enrichment analysis using genes with determined BMC values. No significantly enriched pathways (adjusted *P* < 0.05) were detected for the 6- or 12-week exposure groups when all genes were analyzed together. However, stratification of the transcriptomic data by the direction of expression change revealed significant pathway enrichment among genes showing a decreasing expression trend after 12-week exposure. Among the most significantly enriched pathways were signaling by GPCR, interleukin-10 signaling, dissolution of fibrin clots, synthesis of epoxyeicosatrienoic acids (EET) and dihydroxyeicosatrienoic acids (DHET) and synthesis of (16–20)-hydroxyeicosatetraenoic acids (HETE) (Fig. 8A). The majority of enriched genes were associated with signal transduction and GPCR signaling (Fig. 8A and B).

### Predicted daily oral PFOS intakes required to achieve BMC-equivalent ovarian interstitial fluid concentrations in adult females over a 2-year period

Daily oral PFOS intakes required to reach BMC-equivalent concentrations in ovarian interstitial fluid over a 2-year exposure period were derived for transcriptomic, pathway-level, and apical data following long-term low-level exposure of HGrC1 cells to PFOS.

**Figure 9.**
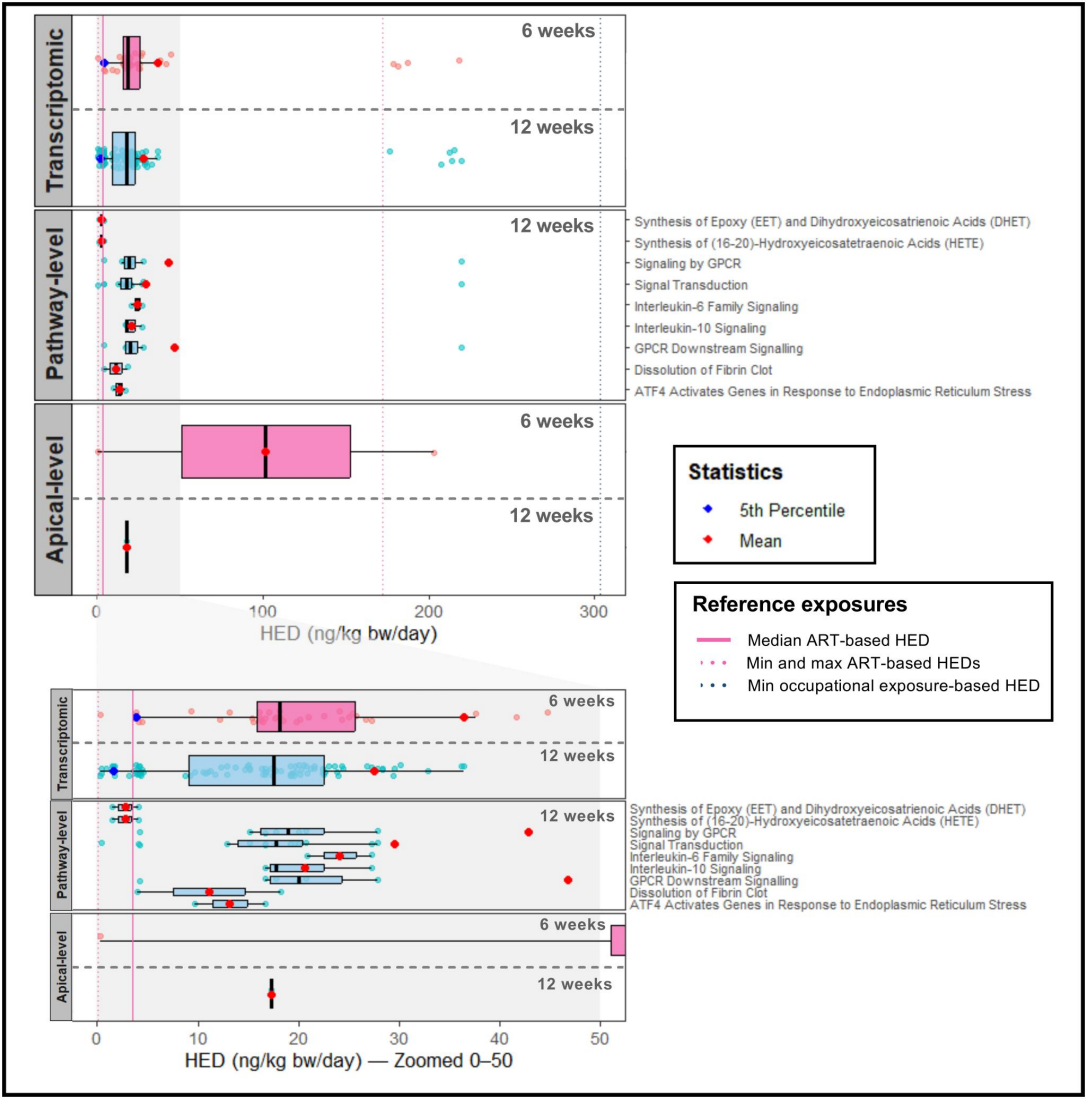
**Human-equivalent doses derived for transcriptomic, pathway-level and apical benchmark concentrations following 6- and 12-week exposures of human granulosa cells (HGrC1) to perfluorooctane sulfonate (PFOS)**. Boxplots summarize the distribution of human-equivalent doses (HEDs, ng/kg body weight (bw)/day): the central line represents the median, the box indicates the interquartile range (25th–75th percentile), and the whiskers show the range excluding outliers. Overlaid jittered points represent individual HEDs.

The model predicted median HEDs of 18.1 ng/kg bw/day (95% CI: 1.1–35.1) and 17.5 ng/kg bw/day (95% CI: 8–27.1) for 6- and 12-week transcriptomic data, respectively, with corresponding 5th percentile HEDs of 3.7 (95% CI: 0.4–9.3) and 1.4 ng/kg bw/day (95% CI: 0.5–3.5). The most sensitive pathways were synthesis of EET and DHET, and synthesis of HETE, with a median HED of 2.8 ng/kg bw/day, whereas interleukin-6 family signaling was the least sensitive with a median of 24.1 ng/kg bw/day. Apical endpoints yielded a wider range of HEDs, from 101.7 ng/kg bw/day (6 weeks) to 17.3 ng/kg bw/day (12 weeks). Notably, higher daily intake was required to achieve BMC associated with changes in estradiol secretion (203 ng/kg bw/day), as opposed to the sub-G_1_ stage of the cell cycle (0.4 ng/kg bw/day) (Fig. 9, Supplementary Table S2).

Reference exposure HEDs corresponding to PFOS concentrations measured in follicular fluid of ART patients were: 3.5 ng/kg bw/day (for average of median concentrations across studies), 0.1 ng/kg bw/day (for minimum across studies) and 172 ng/kg bw/day (for maximum across studies). Reference HEDs derived for median, minimum, and maximum estimated follicular fluid levels in occupationally exposed women (Fu *et al.*, 2016) were 877.3, 303.6, and 13942.7 ng/kg bw/day, respectively (Fig. 9, Supplementary Table S2).

Albumin-corrected BMCs, which potentially produce the same cellular effects in human body (under different albumin levels) as in *in vitro* assays, resulted in higher predicted intake requirements to achieve equivalent ovarian interstitial fluid concentrations (Supplementary Fig. S3, Supplementary Table S3). Median HEDs for the 6- and 12-week transcriptomic data increased to 302 (95% CI: 19–585.1) and 291.8 (95% CI: 132.8–450.8) ng/kg bw/day, respectively, with corresponding 5th percentile HEDs of 61.7 (95% CI: 5.8–154.9) and 22.6 ng/kg bw/day (95% CI: 7.7–58.8). Median HEDs for synthesis of EET and DHET, synthesis of HETE increased to 45.8 ng/kg bw/day, whereas interleukin-6 family signaling reached 401.6 ng/kg bw/day. HEDs derived from apical endpoints ranged from 1694.7 ng/kg bw/day (6 weeks) to 289 ng/kg bw/day (12 weeks). These albumin-corrected values represent an approximately 17-fold increase relative to uncorrected values.

### PFOS bioactivity and human exposure: a BER analysis

**Figure 10.**
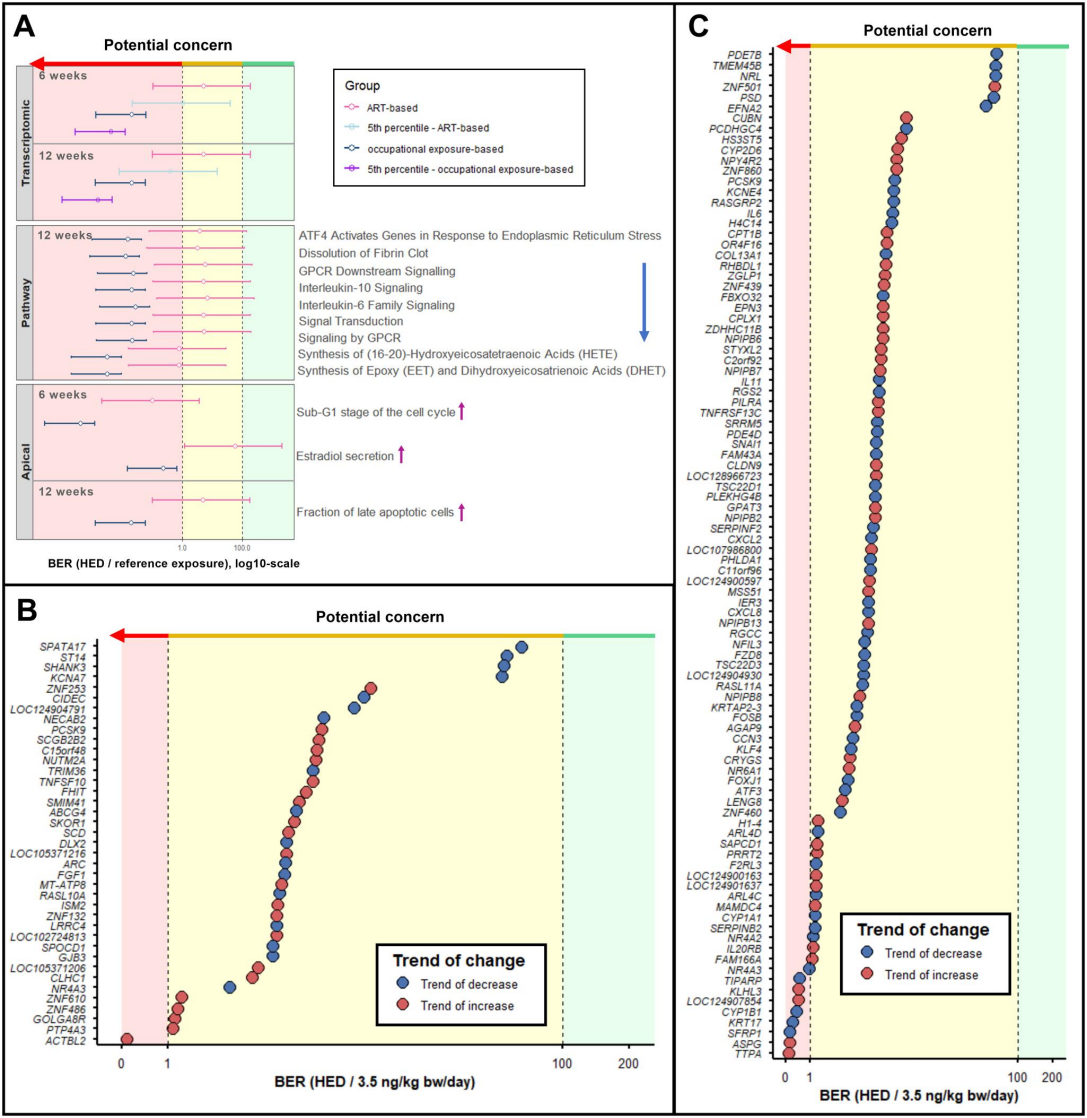
**Bioactivity exposure ratios derived for transcriptomic, pathway-level, and apical endpoints following 6- and 12-week exposures of human granulosa cells (HGrC1) to perfluorooctane sulfonate (PFOS)**. (**A**) Bioactivity exposure ratio (BER) intervals were obtained using the median (circles), minimum, and maximum reference exposures estimated for ART patients and occupationally exposed women. The *X*-axis represents log10-scaled BER values. Distributions of BER values for individual genes, obtained using the median reference exposure estimated for ART patients (3.5 ng/kg body weight (bw)/day), after (**B**) 6-week or (**C**) 12-week exposure to PFOS.

BER values were calculated using HEDs corresponding to the minimum and maximum, as well as the average of median follicular fluid PFOS levels detected across studies in women undergoing ART, which represent the general exposure population. These values are presented as intervals (Fig. 10A) and are provided in Supplementary Table S2. When calculated using the HED corresponding to average of median follicular fluid PFOS concentrations, BER values were below 1 for the most sensitive genes (5th percentile) after 12-week exposure (BER = 0.4) and pathway-level endpoints after 12 weeks, including the synthesis of EET and DHET, and the synthesis of HETE (BER = 0.8). Apical endpoints from the 6-week exposure were evaluated separately, due to unreliable median HED value. The BER interval for the sub-G_1_ fraction of the cell cycle was also lower than 1 (BER = 0.1). BERs for other endpoints remained below 100 (Fig. 10A, Supplementary Table S2), while those calculated using HEDs derived from albumin-corrected BMCs were higher across all endpoints (Supplementary Table S3, Supplementary Fig. S4). After albumin correction, BER values exceeded 1 for all tested endpoints but remained below 100, except for the interleukin-6 family signaling pathway and estradiol secretion.

BERs were also calculated using HEDs corresponding to minimum, maximum, and median estimated follicular fluid PFOS levels in occupationally exposed women. For all endpoints, intervals were below 1 (Fig. 10A, Supplementary Table S2), with most remaining under 1 even after albumin correction (Supplementary Table S3, Supplementary Fig. S4).

To identify genes potentially at highest risk from PFOS exposure, we assessed gene distribution across BERs using the HED for average of median ART-based follicular fluid PFOS concentrations as a reference exposure. After 6 weeks, only one gene (*ACTBL2*) exhibited a BER value below 1, and this gene showed an increasing expression trend (Fig. 10B). After 12 weeks, nine genes had BER values below 1: four genes with trend of expression increase (*TTPA*, *ASPG*, *LOC124907854*, and *KLHL3*) and five genes with trend of expression decrease (*SFRP1*, *KRT17*, *CYP1B1*, *TIPARP*, and *NR4A3*) (Fig. 10C). Following albumin correction, BER values for all genes exceeded 1 at both 6- and 12-week exposure durations (Supplementary Fig. S5).

## Discussion

Reproductive disorders and infertility affect roughly 10–15% women of reproductive age (Infertility Workup for the Women’s Health Specialist, 2019; World Health Organization, 2022). The etiology of ovarian dysfunction is multifactorial and still remains poorly defined. A number of studies have suggested an association between exposure to chemicals, including PFAS and decreased female fertility. Despite the phase-out of some legacy PFAS due to their environmental persistence and adverse health effects, they continue to pollute water and air (Rickard *et al.*, 2022). Epidemiological studies have reported associations between PFOS concentrations in follicular fluid and some infertility factors (Kim *et al.*, 2020). However, direct evidence linking follicular fluid PFOS concentrations to changes in ovarian cell function remains unknown. In this study, we provide compelling and novel evidence by showing that PFOS levels in follicular fluid are sufficient to trigger measurable changes in human granulosa cells.

### Benchmark concentrations

Our results suggest that long-term, low-level PFOS exposure interfered with granulosa cell function. Across multiple apical endpoints, PFOS altered estradiol and progesterone production, the proportions of early and late apoptotic cells as well as necrotic cells, and cell cycle distribution in human granulosa cells. In addition, PFOS induced changes in the expression of several mRNA under both exposure scenarios. However, PFOS exposure caused relatively low cytotoxicity in HGrC1 cells, suggesting that its primary effects may not involve direct cell death. Instead, the observed alterations in hormone secretion, cell cycle regulation, and transcriptomic profiles indicate that PFOS may primarily disrupt signaling pathways regulating granulosa cell function. Such functional disturbances could impair follicular development and ovulation without necessarily causing extensive cellular loss.

To quantify the sensitivity of human granulosa cells to PFOS, we calculated BMC values for all apical and transcriptomic datasets. Median BMCs across most endpoints were between 36.5 and 38.1 nM, indicating that changes in human granulosa cell function occur at low-nanomolar PFOS concentrations. Extending exposure duration did not change the median BMCs, and we observed no consistent shift in relative sensitivity between transcriptomic and apical datasets. The main exception was that apical endpoints after 6 weeks tended to yield slightly higher BMCs than transcriptomic responses.

In a study where 3D *in vitro* mouse ovarian follicles were exposed to PFOS for several days (2–6 days) or for 48 h, BMC values for multiple apical endpoints ranged from 88.9 µM for progesterone production to 2.74 µM for follicle rupture (Pattarawat *et al.*, 2025). When compared to other human-relevant *in vitro* systems, our BMCs are lower than those reported for human liver spheroids, where BMC values were generally in the micromolar range (Reardon *et al.*, 2021; Rowan-Carroll *et al.*, 2021; Addicks *et al.*, 2023). This difference may reflect species-, tissue-, and cell-type specific susceptibility; however, it could also be driven largely by differences in exposure design (long-term exposure vs short-term exposures).

The importance of chronic *in vitro* exposure has also been demonstrated in our previous work with PFOA, where BMCs derived from prolonged exposure were several orders of magnitude lower than those obtained after 48 h in the same cell model (Opacic *et al.*, 2025) and lower than the values reported in liver spheroids after short-term exposure (Reardon *et al.*, 2021; Rowan-Carroll *et al.*, 2021; Addicks *et al.*, 2023). These observations align with recent recommendations emphasizing the value of longer exposure durations and diverse cell-culture systems (e.g. 3D vs 2D) for transcriptomic point-of-departure (POD) derivation and potency ranking (Reardon *et al.*, 2021). Therefore, our experimental approach revealed that human granulosa cells are highly susceptible to PFOS exposure, which raises concern about whether PFOS levels in follicular fluid are sufficient to cause damage observed in *in vitro* experiments.

### HED and BER

To support clinical interpretation, we translated BMCs into HEDs using PBTK modeling and compared the bioactive dose with exposure inferred from PFOS concentrations measured in follicular fluid of women undergoing ART. The resulting BER values provide an estimate on margin between doses of PFOS associated with *in vitro* bioactivity and clinically observed follicular fluid exposure. The median HEDs associated with transcriptomic effects in human granulosa cells were 17.5 and 18.3 ng/kg bw/day for 12- and 6-week exposures, respectively. Using a more conservative metric, the corresponding 5th percentile HEDs for transcriptomic effects were 1.4 and 3.7 ng/kg bw/day. HEDs derived for apical endpoints ranged from 0.4 to 203 ng/kg bw/day, with sub-G_1_ stage of the cell cycle being the most sensitive. At the pathway level, HEDs for PFOS-affected signaling functions ranged from 2.8 to 24.1 ng/kg bw/day, with eicosanoid-related functions, including synthesis of EETs/DHETs and synthesis of HETEs, showing the lowest HED values. These low-effect-equivalent doses translated into BER values near or below 1 for the most sensitive functions indicate that PFOS level in follicular fluid from ART patients may be sufficient to trigger above-mentioned changes in granulosa cells. The magnitude of granulosa cell changes is substantially greater in occupationally exposed populations, indicating that this group may face a higher risk from PFOS exposure.

The HEDs derived here are comparable or lower than the estimates obtained from short-term *in vitro* (ToxCast assays) and animal *in vivo* datasets. For example, a probabilistic PFOS risk assessment integrating human epidemiology, animal studies, and different data from *in vitro* assays on human cells compiling several important human functions such as endocrine, immune, neuro, or PPAR/PXR/RAR receptors estimated a 5th percentile HED of 21.5 ng/kg/day (Chou and Lin, 2020). Another study reported median HED of 80 ng/kg bw/day including the values as low as 0.87 ng/kg/day for developmental diseases or 5.0 ng/kg bw/day for autoimmune thyroid disease (Chen *et al.*, 2022). Our results indicate that transcriptomic or molecular function changes in granulosa cells can occur at similarly low, or lower HEDs, highlighting the functions of human granulosa cells as one of the most sensitive systems to PFOS exposure.

From a reproductive biology perspective, the translation of transcriptomic changes to molecular or biological functions can provide clinically meaningful insight. We showed that PFOS exposure changes EET and DHET- and HETE-related functions. It has been shown that EET may have an important autocrine or paracrine role in regulating ovarian granulosa cell estrogen synthesis (Van Voorhis *et al.*, 1993). Decreased 14,15-EET levels result in accumulation of reactive oxygen species and may impair fertility-related processes (Lin *et al.*, 2025). These two molecular functions show BER < 1, suggesting that clinically observed follicular fluid PFOS concentrations may overlap with effect-equivalent doses for pathways relevant to follicular steroidogenesis and oxidative stress balance. Other human granulosa cell functions such as several interleukins or GPCR signaling pathways show BERs < 10, thereby widening the range of physiological processes that could be affected by PFOS at concentrations measured in follicular fluid. Interleukins modulate ovarian function and play an important role in ovulation (Smolikova *et al.*, 2012), whereas GPCR signaling is indispensable for mediating the actions of gonadotropins and other hormones in the ovary. Together, the number and diversity of granulosa cell functions with low BER values suggest that PFOS concentrations detected in follicular fluid may influence multiple key ovarian processes, raising concern even at environmentally relevant PFOS burdens.

### Gene-level perturbations and candidate biomarkers

We further examined the low-dose tail of the response distribution by focusing on individual gene-level perturbations with particularly low HEDs. This could be important in helping identify candidate biomarkers and high-priority mechanistic targets for validation in clinically relevant samples, such as granulosa or cumulus cells collected during oocyte retrieval. For example, the gene *TTPA* with low BER of 0.1 is best known for regulating vitamin E distribution (Arai and Kono, 2021); however, its role in granulosa-cell physiology is not well characterized. Two genes, *CYP1B1* and *TIPARP*, belonging to the aryl hydrocarbon receptor (AHR) signaling pathway, were both downregulated by PFOS exposure. The characteristics that they exhibit during PFOS exposure make them promising candidate biomarkers and high-priority targets. This is particularly true for *CYP1B1*, since it has been described in human granulosa cells and associated with adverse ovarian phenotypes (Kunitomi *et al.*, 2021). These ovarian phenotypes are more often discussed in the context of increased CYP1B1 activity (Vidal *et al.*, 2005; Chen *et al.*, 2012; Kunitomi *et al.*, 2021), however, reduced expression may also be biologically meaningful. Downregulation of *CYP1B1* together with lower *TIPARP* expression suggests potentially reduced AHR-pathway activity, which could in turn shift the intra-follicular estrogen–metabolite balance (Du *et al.*, 2015), modulate granulosa cell proliferation (Bussmann *et al.*, 2006), and influence oxidative metabolism in granulosa cells (Vidal *et al.*, 2005). Consistent with a potential role of AHR signaling in follicular responsiveness to gonadotropins, reduced AHR activity has also been linked to decreased FSH responsiveness in antral follicles (Hernández-Ochoa *et al.*, 2013). Despite the fact that single gene-level perturbations or dysfunction bears uncertainty whether these PFOS target genes at current follicular fluid concentrations will translate into overt ovarian dysfunction, it could flag high-priority targets, thereby narrowing the margins at the low-dose tail and motivate focused functional validation.

### Strengths and limitations of the study

This study has several important strengths. It combines multiple experimental approaches to comprehensively evaluate the effects of PFOS on human granulosa cells. The study employs long-term, low-level PFOS exposure (up to 12 weeks), a physiologically relevant approach compared with short-term exposures commonly used in *in vitro* studies, enabling the detection of subtle and cumulative cellular and transcriptomic changes. To our knowledge, no previous study has examined long-term, direct PFOS effects in a human granulosa cell line; therefore, this study provides novel insights into ovarian cell susceptibility. The integration of *in vitro* bioactivity data with PBTK modeling allows translation of these findings into HEDs, supporting risk assessment in real-world exposure scenarios. Furthermore, comparison with HEDs derived for PFOS concentrations measured in follicular fluid from women undergoing ART strengthens the clinical relevance of the results. The study also identifies sensitive pathways and candidate genes, which may serve as mechanistic targets for future investigation. By quantitatively linking cellular responses to relevant exposure levels, this study provides a framework for interpreting PFOS reproductive toxicity and supports ongoing efforts to refine health-protective exposure thresholds.

We need to acknowledge several limitations associated with the HED and BER approach used in this study. First, this study utilized human granulosa cells to derive quantitative estimates of bioactivity. While human granulosa cells are the major functional cells in the ovary, they may not fully represent complex cell–cell interactions *in vivo*. Therefore, our bioactivity data may not adequately capture organ-level effects. Second, exposure values used in our study were based on predictions of a PBTK model rather than empirical data, which also bring some degree of uncertainty. Changes in gene expression or apical endpoints observed *in vitro* may not directly translate to equivalent outcomes in animals or humans. In this study, the BMC, which was used for HED and BER calculation, represents a 1 SD of change relative to the control. However, whether a 1 SD change in gene expression *in vitro* corresponds to a similar biological effect *in vivo* remains uncertain. This potential discrepancy in the severity of apical responses between *in vitro* and *in vivo* systems could influence both HED and BER estimations. We would also like to highlight that serum albumin concentrations significantly differ between *in vitro* assays and *in vivo* conditions. We applied a correction factor to predict the concentration of PFOS which would yield the same concentration of free PFOS in humans as in our *in vitro* cell culture system. These predicted values correspond to the bioactive PFOS concentrations in organism yielding a higher BMC and HED values.

## Conclusion

This study shows that the HED inferred from PFOS concentrations measured in follicular fluid of women undergoing ART is above or within a limited margin of the predicted bioactive HEDs derived from both apical and transcriptomic data in human granulosa cells. In addition, the single gene-level perturbations highlight high-priority gene targets and candidate biomarkers that may support future characterization of risk associated with low-level PFOS exposure. These findings raise important concerns regarding potential health risks associated with dietary PFOS exposure for human granulosa-cell and ovarian function. In clinical practice, this evidence supports inclusion of PFOS exposure in the communications regarding environmental risks for women undergoing ART. Reproductive medicine specialists could encourage exposure-reduction strategies including dietary and lifestyle changes aimed to minimize PFOS exposure specifically in patients who may be more vulnerable to ovarian dysfunction. Such approaches may improve ART counseling by integrating environmental health considerations into fertility care.

## Supplementary Material

hoag029_Supplementary_Data

## Data Availability

The data underlying this article will be shared on reasonable request to the corresponding author. The OMICS data are available at GEO (accession number GSE315651).
