# Supplementary material for: Perfluorooctane sulfonate (PFOS) in follicular fluid and human granulosa cell dysfunction: a physiologically based toxicokinetic model translation of long-term low-level in vitro exposure data
Source: Hum Reprod Open. 2026 Apr 7;2026(2):hoag029. doi: 10.1093/hropen/hoag029 (PMC13110862; doi:10.1093/hropen/hoag029)
Supplement: hoag029_Supplementary_Data [file hoag029_supplementary_data.docx]

**SUPPLEMENTARY MATERIAL**

**Perfluorooctane sulfonate (PFOS) in follicular fluid and human granulosa cell dysfunction: a physiologically based toxicokinetic model translation of long-term, low-level *in vitro* exposure data**

Tamara Tomanic^1^, Dragana Samardzija Nenadov^1^*, Sava Radovic Pletikosic^1^, Bojana Stanic^1^, Darija Obradovic^2^, Sasa Lazovic^2^, Nebojsa Andric^1^

^1^Department of Biology and Ecology, Faculty of Sciences, University of Novi Sad, Novi Sad, Serbia

^2^Institute of Physics Belgrade, National Institute of the Republic of Serbia, Belgrade, Serbia

*Correspondence address:

Dragana Samardzija Nenadov, Ph.D.

Department of Biology and Ecology, Faculty of Sciences, University of Novi Sad

Trg Dositeja Obradovica 3, Novi Sad, 21000, Serbia

email: [dragana.samardzija@dbe.uns.ac.rs](mailto:dragana.samardzija@dbe.uns.ac.rs),

ORCID ID: <https://orcid.org/0000-0002-5100-2108>

**SUPPLEMENTARY MATERIALS AND METHODS**

**Functional annotation of distinct expression clusters of differentially expressed genes**

Differentially expressed genes (DEGs) were clustered based on their expression profiles across exposure groups using hierarchical clustering. A dendrogram was generated to visualize the relationships between genes, and the tree was cut to create four distinct expression clusters. Genes within each cluster exhibited similar expression patterns, which were visualized in the heatmap. DEGs belonging to distinct expression clusters were functionally annotated using Gene Ontology (GO) biological process terms. For each cluster of DEGs, GO annotations were retrieved via the org.Hs.eg.db package in R. Pairwise semantic similarity among GO terms within each DEG cluster was computed using the Wang measure implemented in the GOSemSim package (Yu, 2020). The Wang measure quantifies the similarity between GO terms based on their shared semantic structure, allowing us to assess functional relationships among genes within each cluster. Following similarity calculation, GO terms were grouped into functional modules using hierarchical clustering with average linkage. To determine the optimal clustering structure, we applied adaptive tree cutting, which dynamically identifies modules of GO terms that are more closely related to each other. For each functional module, the GO term exhibiting the highest average semantic similarity to all other terms within the module was selected as the representative term. The representative GO terms for each module, along with the number of genes associated with each term, were visualized using ggplot2 for clear presentation of the biological processes most relevant to each DEG cluster. All analyses were performed in R 4.5.2 with Bioconductor 3.22.

**Adjustment of benchmark concentrations for PFOS–albumin binding**

To account for differences in PFOS binding to proteins under *in vitro* and *in vivo* conditions, benchmark concentrations (BMCs) were adjusted for albumin binding. The free fraction of PFOS *in vitro* was estimated to be approximately 5.5% across all tested concentrations using the albumin-binding model described by Kolli (2023), and the accompanying Excel-based calculation tool. Briefly, the association constant (Kₐ) was calculated based on the albumin concentration and the reported fraction of unbound PFOS in human plasma. This Kₐ was then used to estimate the free fraction of PFOS under *in vitro* conditions using the *in vitro* albumin concentration, yielding an unbound fraction across all tested concentrations. BMCs estimated to produce observed cellular effects *in vivo* (referred to as corrected BMCs) were calculated by equating free PFOS concentrations *in vitro* and *in vivo* (BMC_corrected_ = (BMC × 0.055) / 0.0033). These corrected BMCs were subsequently used for HED and BER calculations.

**PBTK model output**

The simulation of chronic PFOS exposure revealed pronounced differences in the distribution of the compound across ovarian sub-compartments. All tissue compartments showed a substantial increase in overall exposure over the two-year period, confirming the strong bioaccumulative behavior of PFOS. The highest AUC values (AUC_tD1–tD2, AUC_tDlast–tDlast, AUC_tEnd) were observed in ovarian blood cells and ovarian plasma, where concentrations reached several orders of magnitude higher levels than in interstitial or intracellular fractions. This pattern reflects the well-known strong affinity of PFOS for blood proteins and its tendency to remain in circulation.

In contrast, the unbound fractions in both interstitial and intracellular spaces remained low (AUC and C_max values in the range of tens of µmol/min/L), consistent with the very small free fraction of PFOS (0.33%) available for diffusion into deeper tissue compartments. Total intracellular concentrations were higher than unbound levels but still markedly lower than those in blood, indicating limited cellular uptake even during prolonged exposure.

Peak concentrations (C_max) followed the same distribution pattern: highest in blood cells and plasma, intermediate in total tissue, and lowest in unbound fractions. The C_trough and C_tEnd values show that a steady state was reached only at the end of the two-year exposure period, characteristic of compounds with an extremely long biological half-life. The t_max across ovarian compartments (corresponding to ~730 days) further confirms that equilibrium was achieved only at the conclusion of the simulation, reflecting the slow redistribution kinetics of PFOS.

Half-life calculations were not possible in most compartments, indicating that no elimination phase was reached during the time frame of the simulation. PFOS continued accumulating until the end of the 2-year exposure window, preventing definition of a terminal slope from which half-life could be estimated. Overall, the toxicokinetic parameters demonstrate the exceptionally high stability and bioaccumulation potential of PFOS in ovarian tissues. Blood compartments serve as the primary reservoir, while the free interstitial and intracellular fractions remain minimal throughout the entire exposure period.

**SUPPLEMENTARY FIGURES**


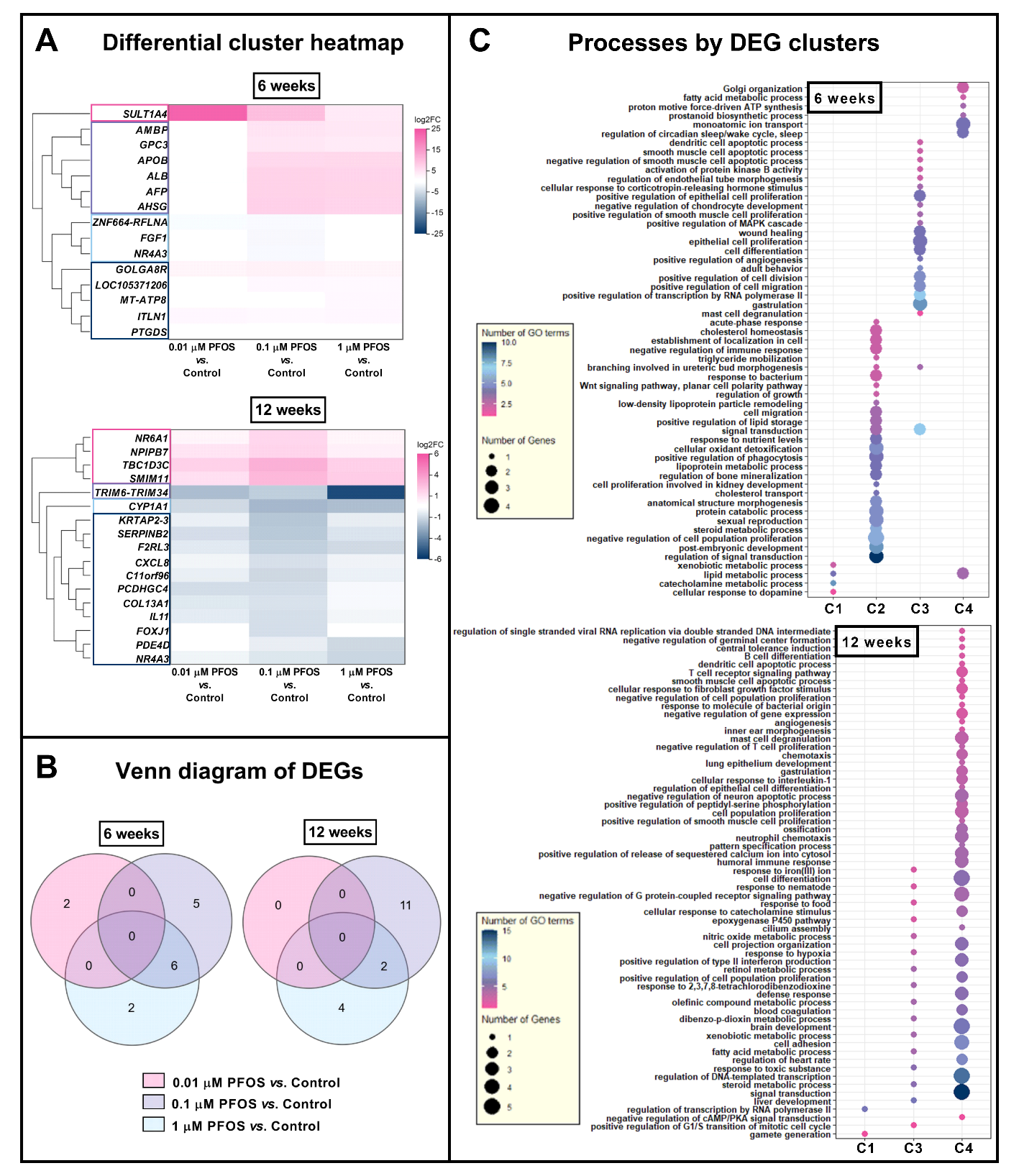
**Supplementary Figure S1. Differential expression and functional clustering of differentially expressed genes in human granulosa cells following long-term, low-level perfluorooctane sulfonate (PFOS) exposure.** HGrC1 cells were exposed for 6 or 12 weeks to 0.01, 0.1, or 1 µM PFOS, or to the vehicle control. (**A**) Differential cluster heatmaps showing distinct expression patterns of differentially expressed genes (DEGs) across exposure concentrations. Cluster 1 (C1) is highlighted in pink, cluster 2 (C2) in purple, cluster 3 (C3) in light blue, and cluster 4 (C4) in dark blue. (**B**) Venn diagrams illustrating overlapping DEGs between exposure groups at each time point. (**C**) Gene Ontology (GO) Biological Process terms significantly associated with each expression cluster.

**
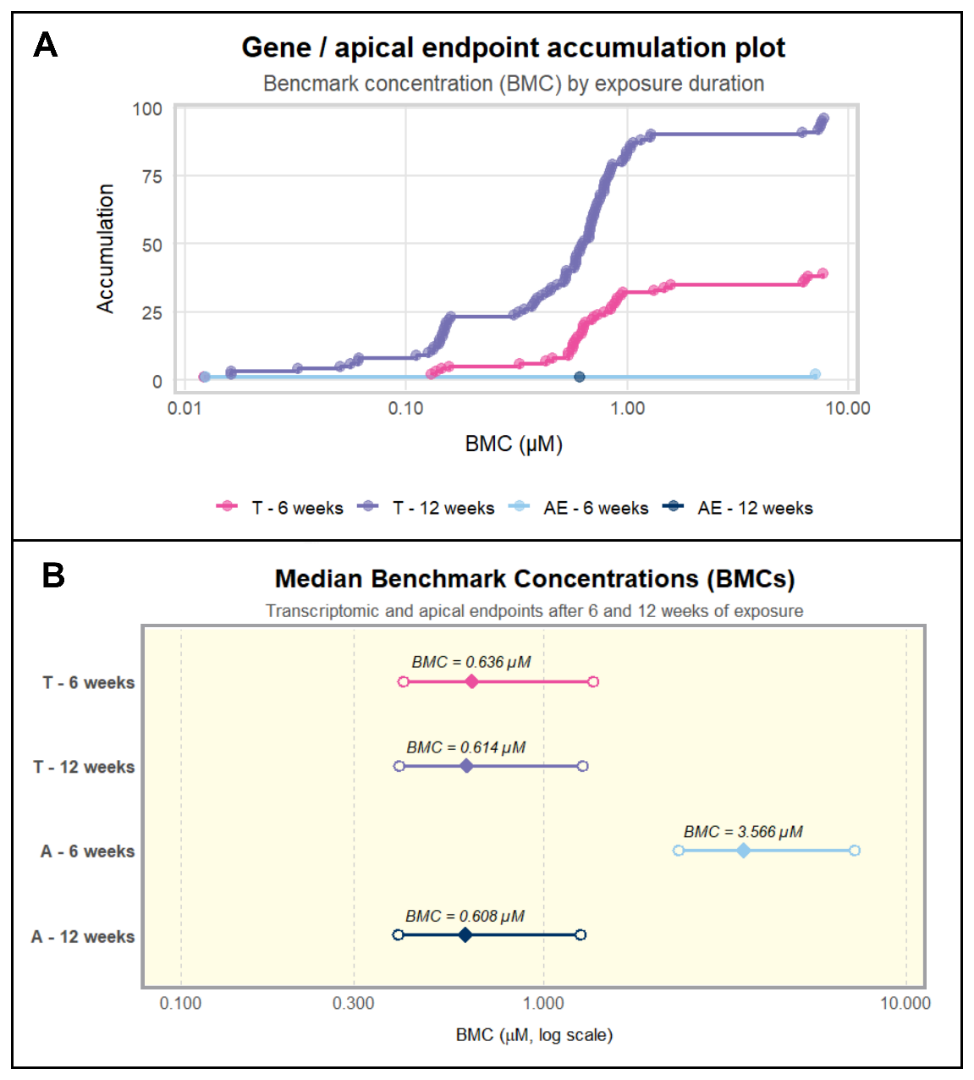
**

**Supplementary Figure S2. Albumin-corrected benchmark concentrations in human granulosa cells following long-term, low-level perfluorooctane sulfonate (PFOS) exposure**. Probes with concentration–response relationships were identified using the Williams Trend Test and the best-fitting models were selected based on the Nested Chi-Square test. (**A**) Accumulation plot of albumin-corrected benchmark concentrations (BMC) for transcriptomic data (T) and apical endpoints (AE) following 6- and 12-week PFOS exposure. (**B**) Range plot illustrating median corrected BMC (diamond) and corrected benchmark concentration lower (BMCL) and upper (BMCU) confidence limits (circles) for transcriptomic (T) and apical (A) data, following 6- and 12-week PFOS exposures.


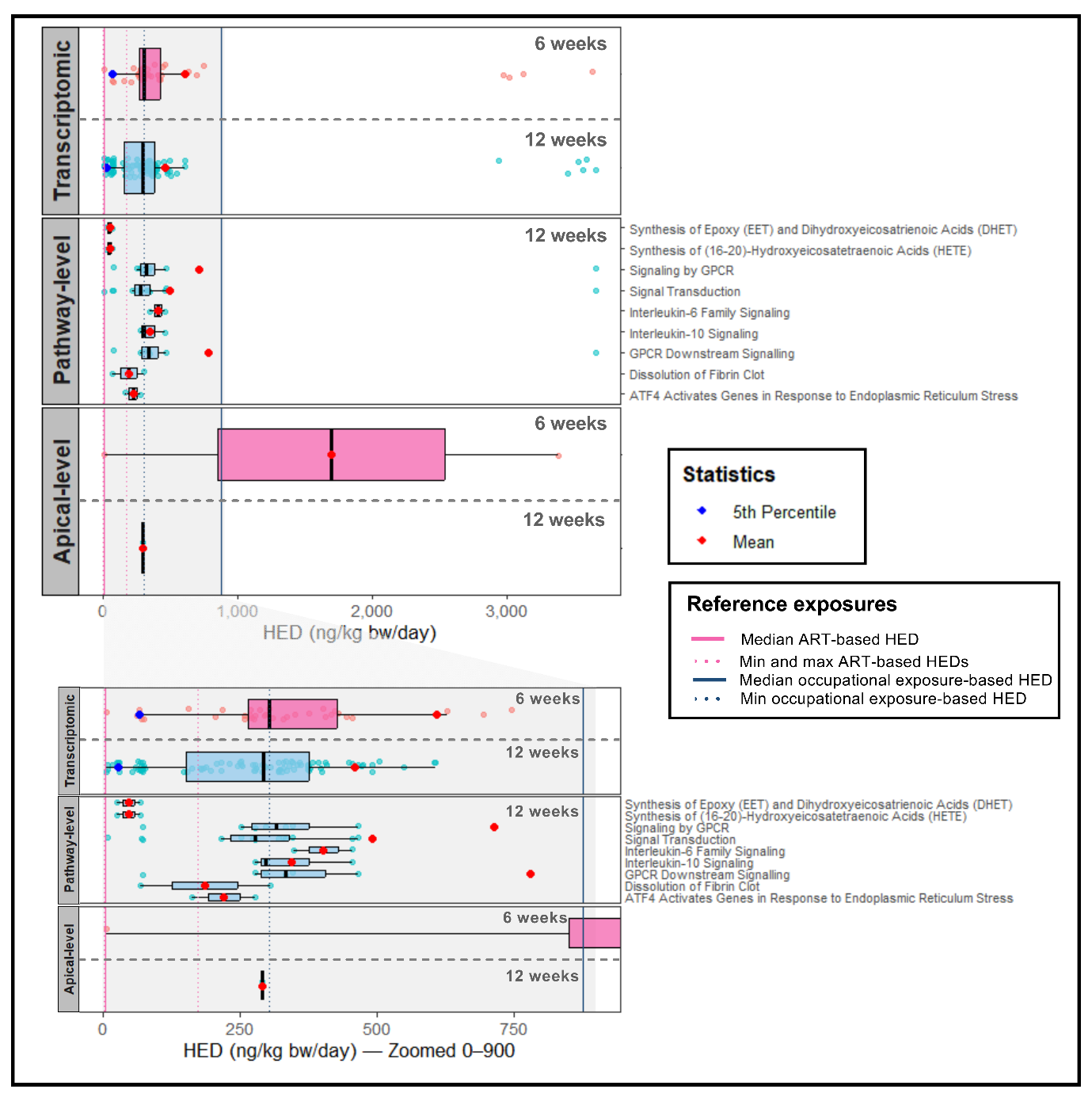


**Supplementary Figure S3.** **Human equivalent doses derived for albumin-corrected transcriptomic, pathway-level, and apical BMC values following 6- and 12-week exposures of human granulosa cells to perfluorooctane sulfonate (PFOS).** Boxplots summarize the distribution of human equivalent doses (HEDs, ng/kg body weight (bw)/day): the central line represents the median, the box indicates the interquartile range (25th–75th percentile), and the whiskers show the range excluding outliers. Overlaid jittered points represent individual HEDs.

**
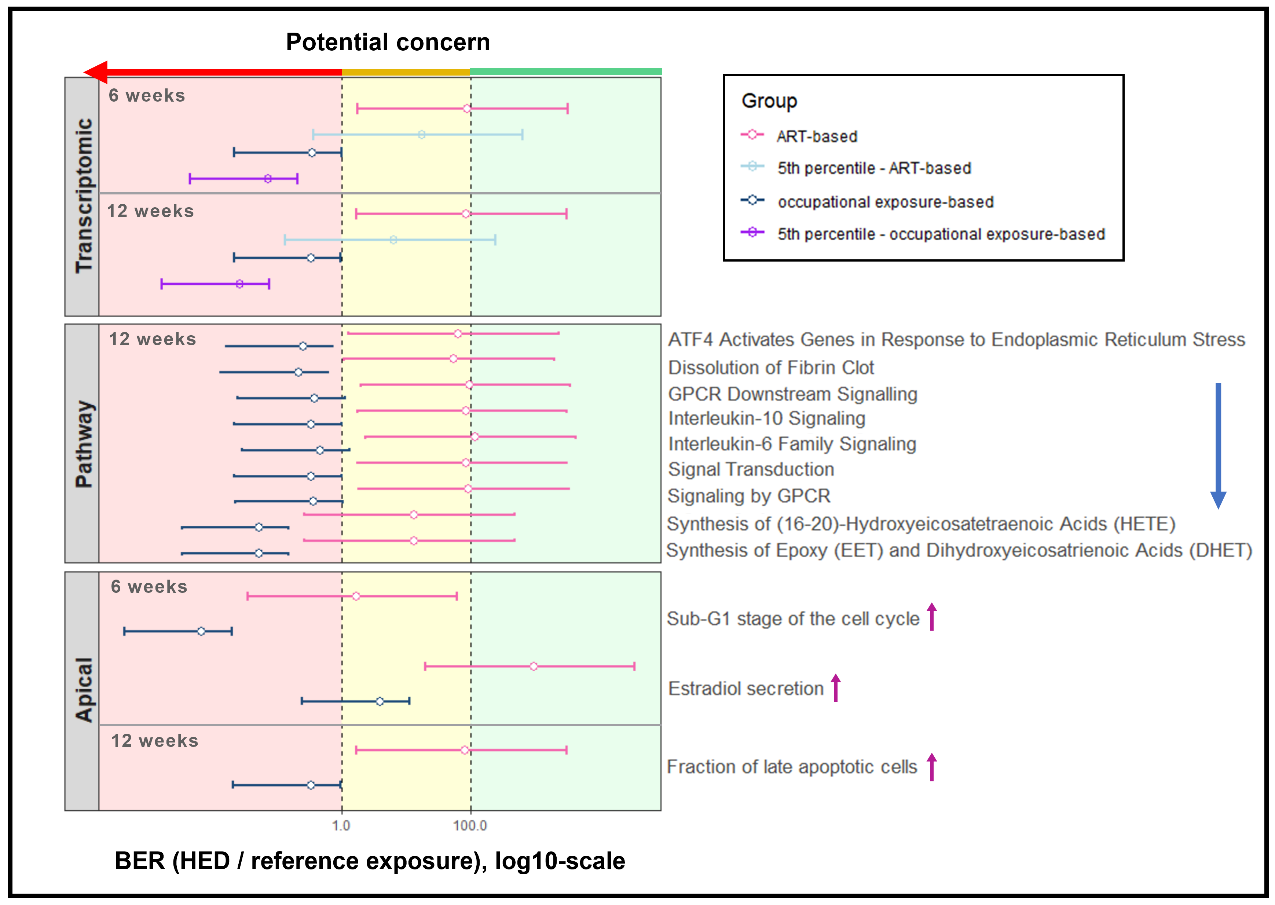
**

**Supplementary Figure S4.** **Albumin-corrected bioactivity exposure ratio values derived for transcriptomic, pathway-level, and apical endpoints following 6- and 12-week exposures of human granulosa cells to perfluorooctane sulfonate (PFOS).** Bioactivity exposure ratio (BER) intervals obtained for corrected human equivalent doses (HEDs) using the median (circles), minimum, and maximum reference exposures for ART patients and occupational scenarios. The X-axis represents log10-scaled corrected BER values.

**
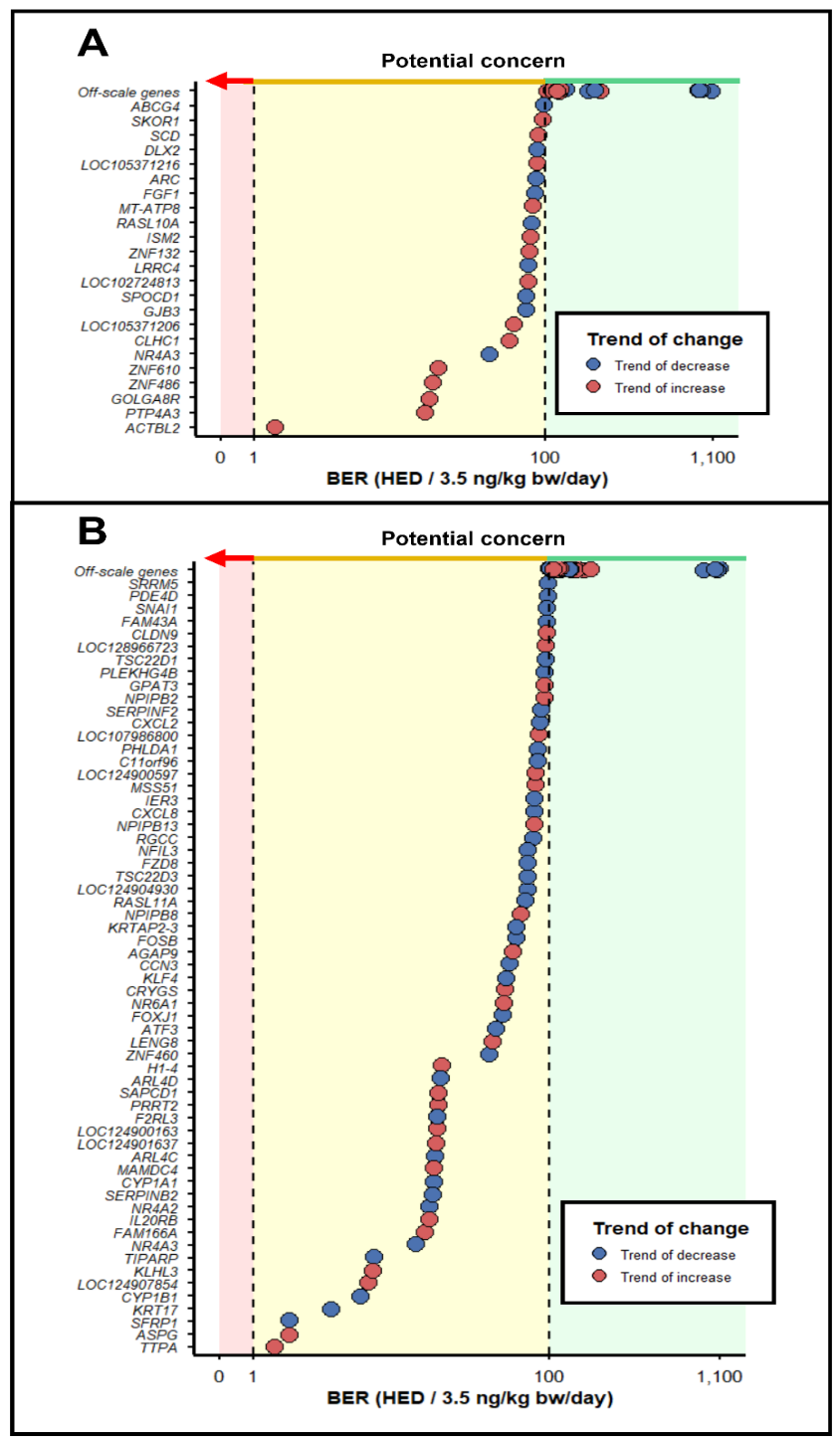
**

**Supplementary Figure S5.** **Distribution of genes across albumin-corrected bioactivity exposure ratio values.** Corrected bioactivity exposure ratio (BER) values were calculated for individual genes following (**A**) 6-week and (**B**) 12-week PFOS exposure, using the median reference exposure estimated for ART patients (3.5 ng/kg body weight (bw)/day).

**SUPPLEMENTARY TABLES**

**Supplementary Table S1.** **Minimum, median and maximum concentrations (ng/mL) of perfluorooctane sulfonate (PFOS) detected in the follicular fluid of ART patients and estimated in occupationally exposed women**.

|  | **PFOS concentrations (ng/mL)** | | |
| --- | --- | --- | --- |
|  | Assisted reproductive technology patients | | |
|  | minimum | median | maximum |
| Bellavia *et al.* (2023) | 0.17 | 2.69 | 15.05 |
| Björvang *et al.* (2022) | 0.171 | 3.54 | 15.051 |
| Boney *et al.* (2026) | 0.15 | 0.787 | 8.83 |
| Clark *et al.* (2024) | 0.46 | 1.3 | 2.3 |
| Heffernan *et al.* (2018) | 0.6 |  | 4.29 |
| Hong *et al.* (2022) | 0.720 | 4.77 | 12.71 |
| Kang *et al.* (2020) | 0.911 | 4.54 | 181 |
| Kim *et al.* (2020) | 0.7 | 4.8 | 22.4 |
| Li *et al.* (2024) |  | 6.04 |  |
| Petro *et al.* (2014) | 0.1 | 7.5 | 30.4 |
| Zeng *et al.* (2023) |  | 1.7 |  |
| Zhang *et al.* (2025) | 0.277 | 1.627 | 19.47 |
|  | Estimated for occupationally exposed women | | |
|  | minimum | median | maximum |
| Fu *et al.* (2016); Zhang *et al.* (2025) | 319.5 | 923.3 | 14,673 |

**Supplementary Table S2.** **Distribution of human equivalent doses and bioactivity exposure ratio values across different endpoints in human granulosa cells following 6- and 12-week perfluorooctane sulfonate (PFOS) exposures.**

|  | |  | |  |  | BER values  (median HED / (median, min-max) reference exposure) | | | |
| --- | --- | --- | --- | --- | --- | --- | --- | --- | --- |
|  | |  | | HED (ng/kg bw/day) | | ART-based | | Occupationally-based | |
| Endpoint | | | | 6-week exposure | 12-week exposure | 6-week exposure | 12-week exposure | 6-week exposure | 12-week exposure |
| Gene | | Median (95% CI) | | 18.1 (1.1-35.1) | 17.5  (8-27.1) | 5.2, 0.1-190.7 | 5, 0.1-184.3 | 0.02, 0.001-0.06 | 0.02, 0.001-0.06 |
|  |  | 5^th^ percentile (95% CI) | | 3.7  (0.4-9.3) | 1.4  (0.5-3.5) | 1.1, 0.02-38.9 | 0.4, 0.008-14.3 | 0.004, 0.0003-0.01 | 0.002, 0.0001-0.005 |
| Pathway median | ∑ | | - | - | - | - | - | - | - |
|  | ↑ | | - | - | - | - | - | - | - |
|  | ↓ | | Synthesis of EET and DHET | - | 2.8 | - | 0.8, 0.02-28.9 | - | 0.003, 0.0002-0.01 |
|  |  |  | Interleukin-10 Signaling | - | 17.8 | - | 5.1, 0.1-187.4 | - | 0.02, 0.001-0.06 |
|  |  |  | Synthesis of HETE | - | 2.8 | - | 0.8, 0.02-28.9 | - | 0.003, 0.0002-0.01 |
|  |  |  | Signaling by GPCR | - | 18.9 | - | 5.4, 0.1-199.1 | - | 0.02, 0.001-0.06 |
|  |  |  | Dissolution of fibrin clot | - | 11.1 | - | 3.2, 0.1-117.1 | - | 0.01, 0.0008-0.04 |
|  |  |  | GPCR downstream signaling | - | 20 | - | 5.8, 0.1-210.7 | - | 0.02, 0.001-0.07 |
|  |  |  | Signal transduction | - | 17.8 | - | 5.1, 0.1-187.4 | - | 0.02, 0.001-0.06 |
|  |  |  | Interleukin-6 family signaling | - | 24.1 | - | 6.9, 0.1-253.6 | - | 0.03, 0.002-0.08 |
|  |  |  | ATF4 activates genes in response to endoplasmic reticulum stress | - | 13.2 | - | 3.8, 0.1-138.9 | - | 0.02, 0.001-0.04 |
| Apical | | Estradiol secretion | | 203 | - | 58.3, 1.2-2136.4 | - | 0.2, 0.02-0.7 | - |
|  |  | Sub-G1 stage of the cell cycle | | 0.4 | - | 0.1, 0.002-3.7 | - | 0.0004, 0.00003-0.001 | - |
|  |  | Fraction of late apoptotic cells | | - | 17.3 | - | 5, 0.1-182.5 | - | 0.02, 0.001-0.06 |
| Range of HEDs derived from PFOS concentrations in follicular fluid of ART patients | | | | 3.5, 0.095 - 172 ng/kg bw/day | | | | | |
| Range of HEDs for estimated PFOS concentrations in follicular fluid under occupational exposure | | | | 877.3, 303.6 – 13942.7 ng/kg bw/day | | | | | |

Bioactivity exposure ratio (BER) values were calculated by dividing human equivalent doses (HED) by median, minimum, and maximum reference exposures in ART patients and occupationally exposed women. Reactome pathway data are provided for all genes (∑), and for subsets of genes exhibiting concentration-dependent increases (↑) or decreases (↓) in expression. bw: body weight; EET: epoxyeicosatrienoic acids; DHET: dihydroxyeicosatrienoic acids; HETE: hydroxyeicosatetraenoic acids.

**Supplementary Table S3.** **Distribution of albumin-corrected human equivalent doses and bioactivity exposure ratio values across different endpoints in human granulosa cells following 6- and 12-week perfluorooctane sulfonate (PFOS) exposures.**

|  | |  | |  |  | BER values  (HED / (median, min-max) reference exposure) | | | |
| --- | --- | --- | --- | --- | --- | --- | --- | --- | --- |
|  | |  | | HED (ng/kg bw/day) | | ART-based | | Occupationally-based | |
| Endpoint | | | | 6-week exposure | 12-week exposure | 6-week exposure | 12-week exposure | 6-week exposure | 12-week exposure |
| Gene | | Median (95% CI) | | 302  (19-585.1) | 291.8 (132.8-450.8) | 86.8, 1.8-3178.3 | 83.8, 1.7-3070.9 | 0.3, 0.02-1 | 0.3, 0.02-1 |
|  |  | 5^th^ percentile (95% CI) | | 61.7  (5.8-154.9) | 22.6,  (7.7-58.8) | 17.7, 0.4-648.8 | 6.5, 0.1-238.2 | 0.07, 0.004-0.2 | 0.03, 0.002-0.07 |
| Pathway median | ∑ | | - | - | - | - | - | - | - |
|  | ↑ | | - | - | - | - | - | - | - |
|  | ↓ | | Synthesis of EET and DHET | - | 45.8 | - | 13.1, 0.3-481.6 | - | 0.1, 0.003-0.2 |
|  |  |  | Interleukin-10 Signaling | - | 296.8 | - | 85.3, 1.7-3123.8 | - | 0.3, 0.02-1 |
|  |  |  | Synthesis of HETE | - | 45.8 | - | 13.1, 0.3-481.6 | - | 0.1, 0.003-0.2 |
|  |  |  | Signaling by GPCR | - | 315.3 | - | 90.6, 1.8-3317.8 | - | 0.4, 0.02-1 |
|  |  |  | Dissolution of fibrin clot | - | 185.4 | - | 53.3, 1.1-1951.1 | - | 0.2, 0.01-0.6 |
|  |  |  | GPCR downstream signaling | - | 333.7 | - | 95.9, 1.9-3511.7 | - | 0.4, 0.02-1.1 |
|  |  |  | Signal transduction | - | 296.8 | - | 85.3, 1.7-3123.8 | - | 0.3, 0.02-1 |
|  |  |  | Interleukin-6 family signaling | - | 401.6 | - | 115.4, 2.3-4226 | - | 0.5, 0.03-1.3 |
|  |  |  | ATF4 activates genes in response to endoplasmic reticulum stress | - | 219.9 | - | 63.2, 1.3-2314.3 | - | 0.3, 0.02-0.7 |
| Apical | | Estradiol secretion | | 3383.4 | - | 972, 19.7-35606.8 | - | 3.9, 0.2-11.1 | - |
|  |  | Sub-G1 stage of the cell cycle | | 5.9 | - | 1.7, 0.03-62.1 | - | 0.007, 0.0004-0.02 | - |
|  |  | Fraction of late apoptotic cells | | - | 289 | - | 83, 1.7-3041 | - | 0.3, 0.02-1 |
| Range of HEDs derived from PFOS concentrations in follicular fluid of ART patients | | | | 3.5, 0.095 - 172 ng/kg bw/day | | | | | |
| Range of HEDs for estimated PFOS concentrations in follicular fluid under occupational exposure | | | | 877.3, 303.6 – 13942.7 ng/kg bw/day | | | | | |

Bioactivity exposure ratio (BER) values were calculated by dividing human equivalent doses (HED) by median, minimum, and maximum reference exposures in ART patients and occupationally exposed women. Reactome pathway data are provided for all genes (∑), and for subsets of genes exhibiting concentration-dependent increases (↑) or decreases (↓) in expression. bw: body weight; EET: epoxyeicosatrienoic acids; DHET: dihydroxyeicosatrienoic acids; HETE: hydroxyeicosatetraenoic acids.

**Supplementary References**

Björvang RD, Hallberg I, Pikki A, Berglund L, Pedrelli M, Kiviranta H, Rantakokko P, Ruokojärvi P, Lindh CH, Olovsson M, *et al.* Follicular fluid and blood levels of persistent organic pollutants and reproductive outcomes among women undergoing assisted reproductive technologies. *Environ Res* 2022;**208**:112626.

Hong A, Zhuang L, Cui W, Lu Q, Yang P, Su S, Wang B, Zhang G, Chen D. Per- and polyfluoroalkyl substances (PFAS) exposure in women seeking in vitro fertilization-embryo transfer treatment (IVF-ET) in China: Blood-follicular transfer and associations with IVF-ET outcomes. *Sci Total Environ* 2022;**838**:156323.

Kang Q, Gao F, Zhang X, Wang L, Liu J, Fu M, Zhang S, Wan Y, Shen H, Hu J. Nontargeted identification of per- and polyfluoroalkyl substances in human follicular fluid and their blood-follicle transfer. *Environ Int* 2020;**139**:105686.

Kolli AR. Deriving protein binding‐corrected chemical concentrations for in vitro testing. *Clin Transl Sci* 2023;**16**:2123–2129.

Yu G. Gene Ontology Semantic Similarity Analysis Using GOSemSim. In Kidder BL, editor. *Stem Cell Transcr Netw* 2020;**2117**:207–215.
